# In-ear EEG wearables for brain activity assessment and cognitive rehabilitation: the emerging role of multimodal embedded intelligence

**DOI:** 10.3389/fnhum.2026.1793705

**Published:** 2026-04-20

**Authors:** Asma Channa, Herbert F. Jelinek, Abdelkader Nasreddine Belkacem, Mohamed Atef, Ibrahim (Abe) M. Elfadel

**Affiliations:** 1 Center for Cyber Physical Systems and Department of Computer and Information Engineering, Khalifa University, Abu Dhabi, United Arab Emirates; 2 Department of Clinical Sciences, Khalifa University, Abu Dhabi, United Arab Emirates; 3 Department of Computer and Network Engineering, United Arab Emirates University, Abu Dhabi, United Arab Emirates; 4 Department of Electrical and Communication Engineering, United Arab Emirates University, Abu Dhabi, United Arab Emirates

**Keywords:** brain activity, cognitive monitoring, cognitive rehabilitation, EEG, embedded intelligence, in-ear wearable device, stimulation, system design

## Abstract

This literature review critically examines the design, validation, and application of non-invasive in-ear electroencephalography (ear-EEG) systems as emerging wearable platforms for long-term neurophysiological monitoring and intervention. Following PRISMA guidelines, studies published between 2010 and 2025 were systematically selected from four major databases and organized into four thematic domains: in-ear wearable system design and validation, multimodal sensing and stimulation, embedded intelligence, and brain-state monitoring and rehabilitation. The review focuses exclusively on wearable, ear-centered EEG technologies, explicitly excluding cochlear implants and other invasive or behind-the-ear systems. We analyze key engineering challenges unique to ear-EEG, including electrode placement constraints, mechanical–electrical coupling, motion robustness, power efficiency, and long-term wearability. The review highlights a growing transition toward compact, wireless ear-EEG systems with on-device signal processing and embedded machine learning, enabling real-time brain-state estimation under ambulatory conditions. Multimodal integration, combining ear-EEG with complementary sensors such as EOG, inertial units, and cardiovascular signals is shown to improve artifact awareness, contextual interpretation, and closed-loop capability. Beyond summarizing existing technologies, this review identifies critical gaps limiting clinical translation, including the lack of standardized validation protocols, limited embedded autonomy, and underexplored closed-loop neurofeedback and neuromodulation architectures. By synthesizing advances across hardware design, signal processing, and intelligent system integration, this work provides a systems-level roadmap for the future development of wearable, intelligent, and clinically robust ear-EEG platforms for mental health, neurorehabilitation, and continuous brain monitoring.

## 1 Introduction

Wearable technology provides the possibility of prolonged monitoring of physiological parameters, with potential applications in healthcare, sports science and medicine, and in extreme environments. Among emerging wearable technologies, in-ear devices or earables possess technical advantages for long-term monitoring, because of their non-invasivity, non-obtrusivity, and stable adhesion, as well as physiological advantages related to the proximity of the ear to the body trunk and the shared vasculature between the ear and the brain as described by Masè et al. (2020). It can detect a variety of biosignals noninvasively, even better than the wrist because it is closer to the heart and brain and less susceptible to motion artifacts (Azudin et al., 2023; Xu et al., 2024). Moreover, the electrodes are under pressure from the tight fit of an earpiece inside the ear canal, which guarantees stable electrode placements and somewhat eliminates motion aberrations that usually obfuscate the signal quality in a traditional electroencephalograph (EEG) systems. Due to these benefits, ear EEG devices have gained popularity as a potentially useful tool for the continuous assessment of brain activity outside the laboratory or clinical settings as highlighted by Joyner et al. (2024).

**Figure 1:**
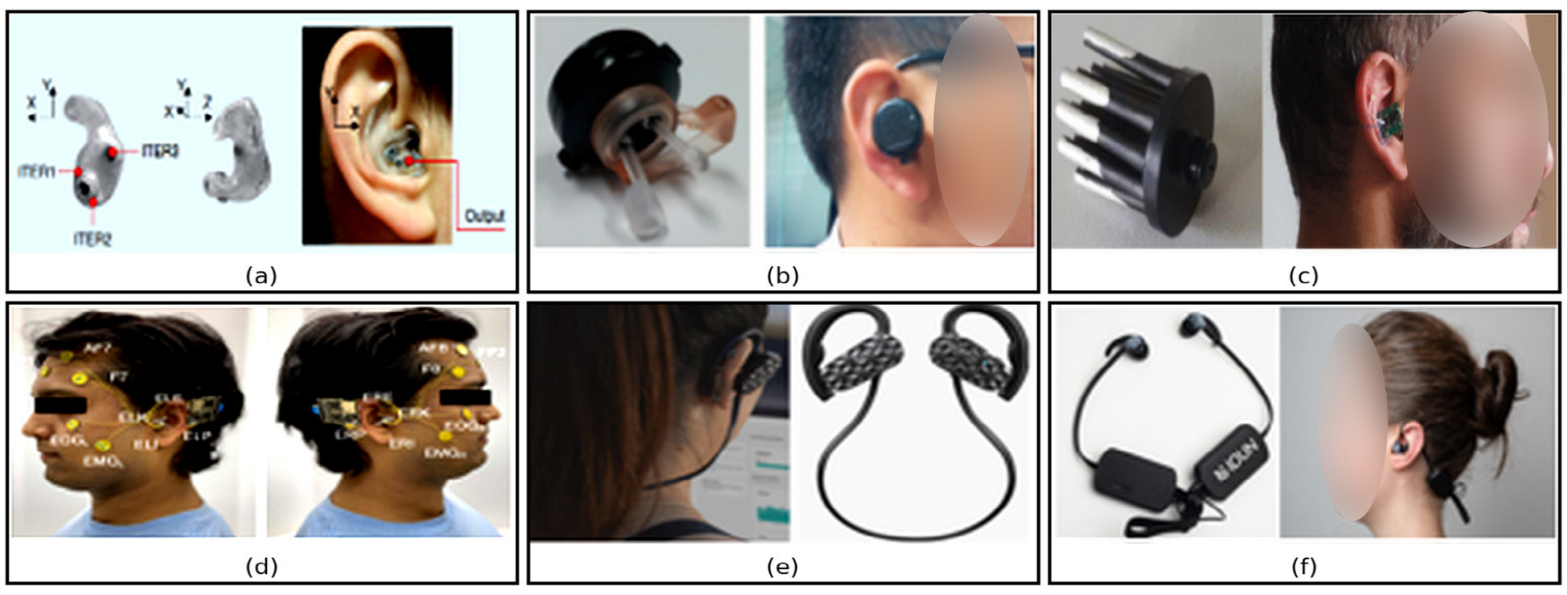
Representative examples of ear-centered EEG and related wearable systems. **(a)** The first in-ear EEG prototype introduced by Looney et al. (2011). Reproduced with permission. **(b)** An in-ear wearable pressure-sensing device illustrating early ear-canal instrumentation concepts (Park et al., 2015). **(c)** A wireless ear-EEG system used to record brain responses to external auditory stimuli (Guermandi et al., 2022). Reproduced with permission. **(d)** An untethered subject equipped with in-ear EEG electrodes and surface reference electrodes supported by dual wearable data acquisition units (Paul et al., 2023). Reproduced with permission. **(e)** The Emotiv MN8, a commercially available two-channel EEG headset with ear-adjacent electrodes, included for comparison Emotiv (2025). Reproduced with permission. **(f)** The IDUN Guardian earbuds, representing a contemporary commercial ear-EEG platform for long-term neurophysiological monitoring (IDUN Technologies, 2025). Reproduced with permission.

The earliest in-ear EEG recording equipment was introduced by Looney et al. (2012), as shown in Figure 1a. Subsequently, several innovative designs of ear-centered devices have been proposed and validated. The expansion of these ear-EEG systems has been influenced by their application in various domains. For example, Park et al. (2015) developed a wearable in-ear sensor to measure Ear Pulse Waves (EPW), as shown in Figure 1b. Consequently, the researchers in Guermandi et al. (2022) designed a 600-hour battery-operated wireless system for EEG acquisition and processing in an earbud form factor, as shown in Figure 1c. In Paul et al. (2023) the authors presented a portable, user-generic, low profile ExG gadget with a variety of biosensing features in the ear and in the body, as shown in Figure 1d. In terms of industry, both established and start-up companies have taken initiatives to develop in-ear devices for mental health monitoring. Emotiv (2025) and IDUN Technologies (2025) have developed hassle-free ear-EEG systems in the form of headphones and earbuds, as shown in Figures 1e, f. This shows that in-ear EEG technology has the potential to contribute to the development of new monitoring and assessment procedures for various clinical conditions. However, it also presents challenges that need to be addressed. These challenges include material selection, biosignal quality, artifact removal, ultralow-power electronics, and ergonomic system design. Although several wearable in-ear EEG devices have hit the market such as NAOX Technologies (2025), these technologies still require independent validation against clinical-grade, gold-standard devices. In this context, our review focuses on the design and performance of in-ear EEG technologies, with particular emphasis on their emerging role in brain activity monitoring and cognitive rehabilitation. Unlike conventional EEG caps, in-ear platforms increasingly support multimodal biosensing and cross-signal integration, while embedding intelligence directly on microcontrollers and system-on-chip (SoC) platforms. This circuits-and-systems perspective is critical, as advances in low-noise analog front-ends (AFEs), power-efficient SoCs, and synchronized firmware pipelines are redefining the balance between device miniaturization, computational autonomy, and long-term wearability.

This survey makes four core contributions. First, it offers a systematic overview of system design, addressing challenges such as ear-shape variability, electrode integration (e.g., sintered Ag/AgCl for low-noise performance), and energy supply innovations including motion-based energy harvesting. Second, it evaluates multimodal sensing and stimulation, integrating EEG with ECG, sweat glucose, and inertial signals, and examining electrical, acoustic, and magnetic stimulation for enhanced neuroplasticity. Third, it highlights the role of embedded intelligence, where modern SoCs and low-power microcontrollers enable real-time analysis through lightweight machine learning methods enabling real-time EEG-based inference under embedded constraints (Nakamura et al., 2017; Jeong and Jeong, 2020). Finally, it emphasizes clinical translation and rehabilitation outcomes, underscoring how in-ear EEG wearables have shown promise in improving speech, pain management, attention, and motor function recovery.

Taken together, these contributions establish a roadmap for the advancement of in-ear EEG wearables, showing how circuit and system innovation, multimodal integration, and embedded intelligence converge to enable next-generation platforms for monitoring and rehabilitation of brain health.

The remainder of the article is organized as follows. Section 2 outlines the methodology we have employed to identify the scope of this review. Section 3 outlines the design and validation of in-ear wearables. Section 4 reviews the sensing and stimulation modalities of ear devices, while Section 5 explores their current use to assess and rehabilitation brain activity. Section 6 is focused on the instrumentation of in-ear wearables with embedded medical intelligence to improve patient outcomes. Section 8 addresses methodological biases in the surveyed literature and discusses key limitations at the device level. The article is concluded in Section 9.

## 2 Review methodology

This section explains the research methodology used for this systematic review. First, PROSPERO (2025) is adopted to identify that a systematic review on this topic is not already available to avoid duplication of work. Then the preferred reporting items for systematic reviews and meta-analyses (PRISMA) guidelines are followed to perform this review (Liberati et al., 2009). To ensure comprehensive literature retrieval, we adopted the following keyword query: *(Ear AND EEG) OR (Ear AND Electroencephalography) OR (Ear AND Electroencephalogram) OR (In-Ear wearable AND EEG) OR (In-Ear device AND Electroencephalography) OR (In-Ear wearable AND Electroencephalogram) OR (Inner-Ear wearable AND EEG) OR (Inner-Ear device AND Electroencephalography) OR (Inner-Ear wearable AND Electroencephalogram)*. This query was designed to capture relevant studies related to in-ear EEG devices and their applications. With this keyword query, the search was performed over the period of 2010–2025 in four well-known datasets: Web of Science, Scopus, IEEE Xplore, and PubMed. A total of 2,202 records were identified, of which 829 duplicates were removed. Their duplication was due to their appearance in multiple database sources.

**Figure 2:**
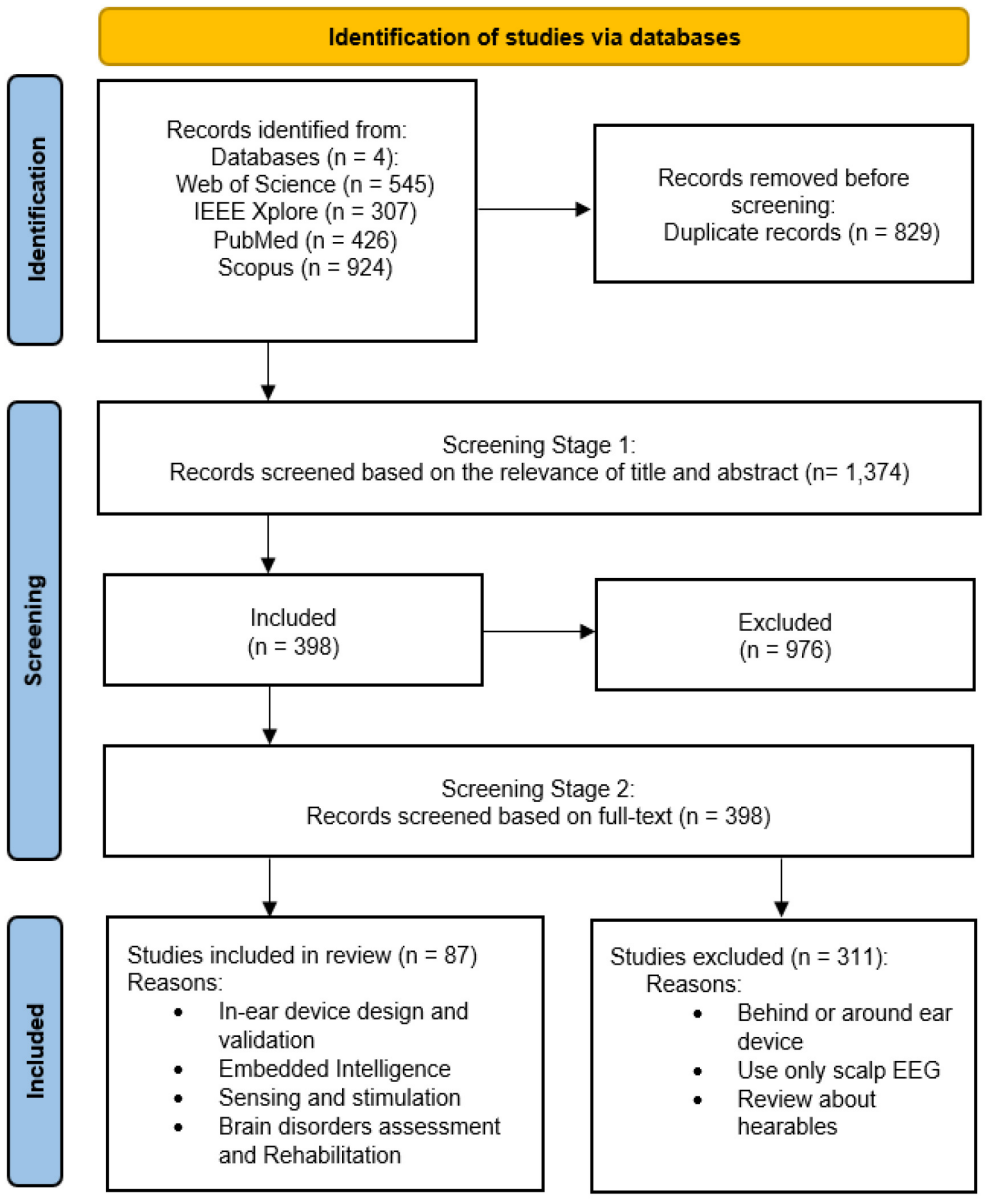
PRISMA chart for methodical paper selection and evaluation of quality.

The next step was to perform several stages of the screening of the remaining records. In the first screening stage, the relevance of the articles was checked according to their titles and abstracts. The outcome of this stage was to keep 398 articles, and the rest were excluded. In the second stage of screening, relevance is further checked on the basis of the full text, with each article classified according to the following categories: in-ear wearable device design and validation, embedded intelligence, sensing and stimulation, multimodal physiological signal analysis, and brain activity assessment and rehabilitation. Article duplicates and articles outside of the classification categories were eliminated. The complete selection methodology that resulted in the final database is summarized in Figure 2.

**Figure 3:**
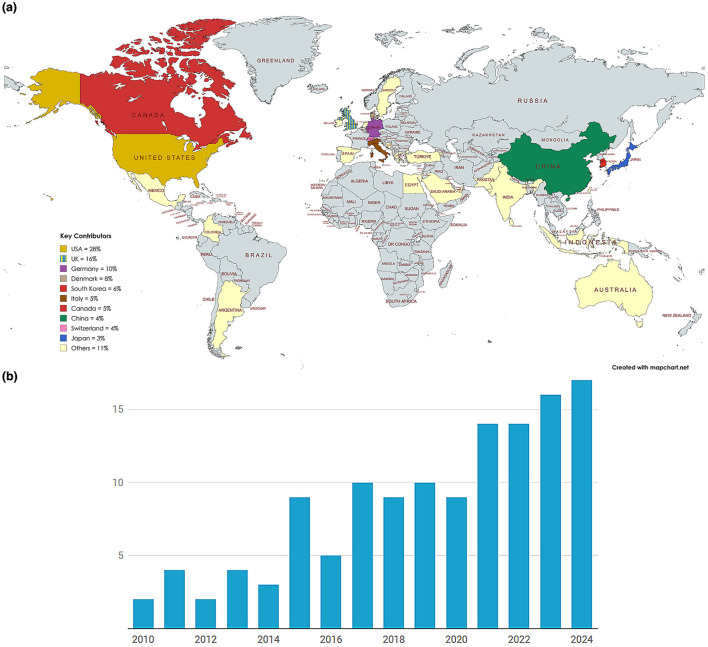
Overview of global and temporal trends in in-ear EEG research. **(a)** Countries contributing to in-ear EEG studies based on first author affiliation. **(b)** Number of peer-reviewed publications related to in-ear EEG devices from 2010 to 2024. A notable increase in research activity is observed in the past five years, reflecting growing interest in wearable neurotechnology and its healthcare applications. **(a)** Geographic distribution of research on in-ear EEG devices. **(b)** Annual number of publications on in-ear EEG (2010–2024).

This review synthesizes 87 peer-reviewed publications (2010–2025) on in-ear wearable devices, selected through a systematic search of IEEE Xplore, PubMed, and Scopus databases. The dataset reveals a strong concentration in North America (USA: 28%, Canada: 5%) and Europe (UK: 16%, Germany: 10%, Denmark: 8%), reflecting established biomedical engineering hubs as shown in Figure 3a. Emerging contributions from Asia (South Korea: 6%, China: 4%, Japan: 3%) highlight the growing investment in hearable technologies. In particular, 11% of the studies involved multi-national collaborations. The bar graph in Figure 3b illustrates the yearly number of publications on in-ear EEG devices from 2010 to 2024. Research activity remained relatively low until 2014, followed by gradual growth between 2015 and 2020. A noticeable surge occurred from 2021 onward, peaking in 2024. The consistent upward trend, especially in recent years, demonstrates growing recognition of in-ear EEG systems as promising tools for real-world, unobtrusive neuromonitoring applications.

## 3 In-ear wearable system design and validation

Compared to scalp EEG, the signal recorded within the ear usually has a smaller amplitude (Moumane et al., 2024). This is probably because the recording sites inside the ear are farther away from the brain's producing sources, and the electrodes employed for recording have different electrical and geometric characteristics. The recording of bioelectrical signals from electrodes placed on the skin surface depends critically on the electrode-skin interface (ESI). Ear EEG devices usually cover a lower spatial extent than a standard scalp EEG device, with most of the recorded activities originating from temporal regions as described by Kappel et al. (2018). Therefore, to compensate for spatial information loss, a correct location and a firm electrode-skin contact are important. The readout electronics pose the second challenge for in-ear device design and validation. They must be hidden (perhaps completely in the ear), low-power (to ensure a long enough battery life), and have on-board computation capabilities (to process on-the-edge, avoiding the need for a companion device and continuous data transmission to such device).

### 3.1 Electrode placement and signal acquisition

**Figure 4:**
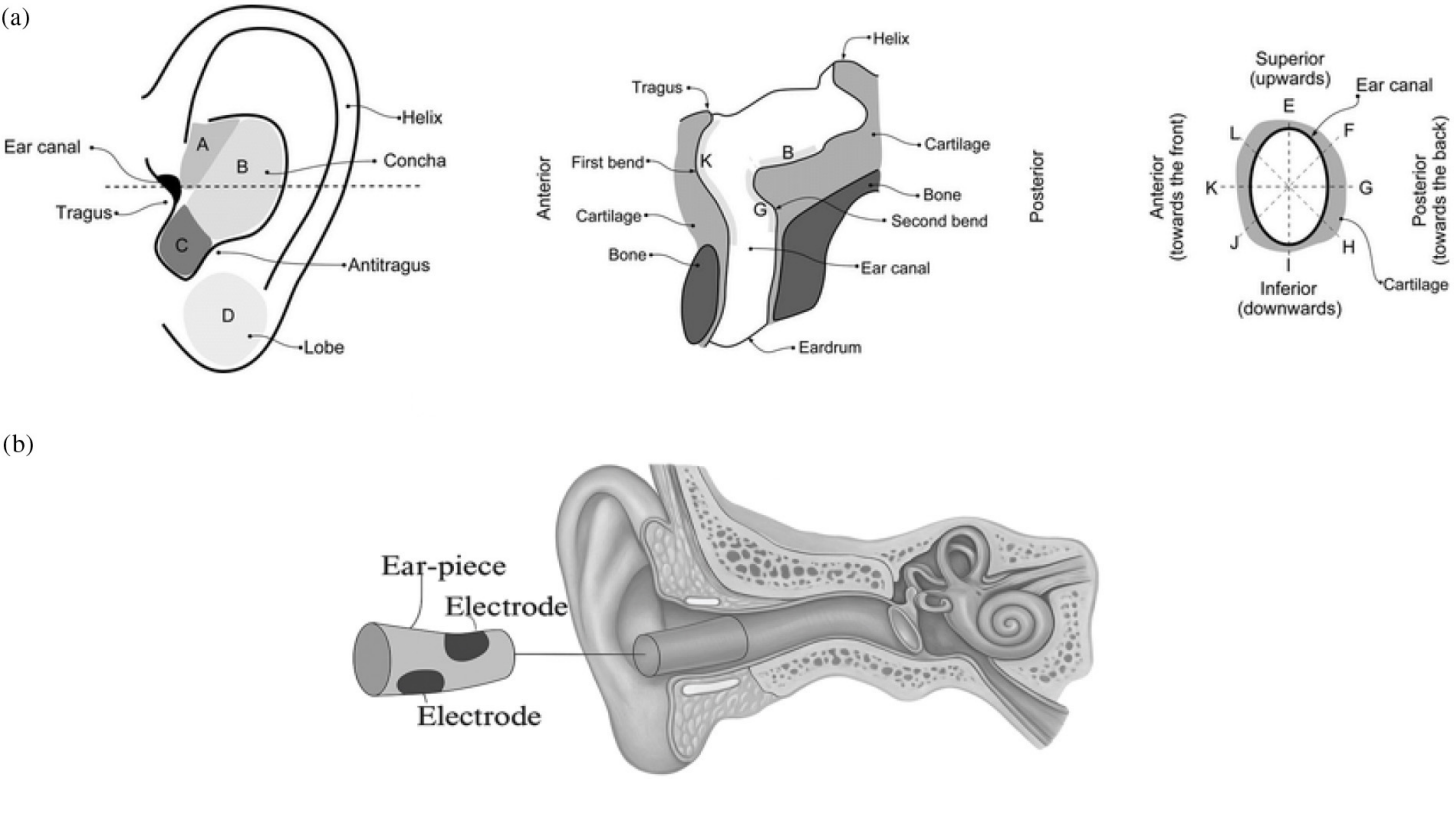
Electrode configurations in in-ear EEG systems Kidmose et al. (2013b). **(a)** External ear placements (e.g., concha, tragus, and antitragus). **(b)** Canal-based placement using a custom-molded ear-piece with embedded electrodes. **(a)** External anatomical landmarks used for electrode placement. **(b)** Cross-sectional view showing in-ear canal electrode placement. Reproduced with permission.

Electrode placement is a critical design consideration in in-ear and around-ear EEG systems, directly influencing signal amplitude, artifact susceptibility, spatial specificity, and long-term wearability. As illustrated in Figure 4, electrodes can be positioned at several anatomical sites—including the concha, tragus, antitragus, external canal entrance, and the deeper ear canal—each offering advantages for specific sensing tasks. External placements, such as those in Figure 4a, position electrodes on the concha, tragus, or antitragus; these sites are easily accessible, support integration with standard ear-worn form factors, and have been widely used to record auditory evoked potentials, alpha-band activity, and responses to transcutaneous auricular vagus nerve stimulation (taVNS) (Kidmose et al., 2013b; Mikkelsen et al., 2015). Concha and tragus-based electrodes typically exhibit higher signal amplitude than deeper in-canal electrodes due to larger electrode–skin contact area, but they remain more susceptible to jaw movement and facial muscle artifacts. Canal-based configurations, shown in Figure 4b, embed electrodes inside a custom-molded earpiece positioned deeper in the ear canal, and literature consistently reports several advantages of this approach, including higher mechanical stability due to tight canal fit (supporting motion-robust recording during walking or exercise (Looney et al., 2012; Goverdovsky et al., 2017) improved SNR (signal-to-noise ratio) for low-frequency components, and better comfort for multi-hour or overnight recordings, demonstrated in sleep staging studies by Moumane et al. (2024) and Nakamura et al. (2019). Although canal-EEG often exhibits slightly lower amplitude than concha-EEG because of smaller contact area and greater distance from cortical sources, it remains effective for applications such as sleep staging, workload monitoring, and sustained alpha recordings where stability is more important than amplitude. Across studies, a clear pattern emerges: concha and tragus electrodes provide higher-amplitude EEG but are more prone to artifacts, whereas in-canal electrodes offer lower amplitude but substantially greater motion robustness ideal for long-term or ambulatory EEG monitoring. These findings highlight that no single placement is universally superior, and electrode selection must align with the intended sensing task—higher-amplitude, event-related EEG benefits from external auricular electrodes, whereas long-duration, motion-tolerant monitoring favors canal-based systems—and anatomical variability continues to motivate improved custom earpiece fabrication and flexible electrode arrays. These trade-offs, summarized in Table 1, underline the absence of a universally optimal electrode location and motivate task-specific electrode placement strategies in ear-EEG system design.

**Table 1:** Summary of lessons learned from literature on ear-based EEG electrode placements.

Location	Advantages	Limitations/notes	Key references
**Concha**	Large contact area; relatively higher signal amplitude; good sensitivity to temporal cortex activity; widely used in early ear-EEG and taVNS studies.	Susceptible to jaw motion and facial EMG; stability degrades under strong head movement.	Mikkelsen et al. (2015); Bleichner and Debener (2017)
**Tragus/antitragus**	Easily accessible; compatible with earhooks and earbud shells; commonly used in auricular stimulation and ear-adjacent EEG.	Moderate EMG and motion contamination; signal quality depends strongly on placement geometry.	Konakoğlu et al. (2023); Badran et al. (2022)
**Canal Entrance**	Easier fitting than deep canal; compatible with standard earbud tips; moderate mechanical stability.	More motion-sensitive than deep canal; higher inter-subject impedance variability.	Goverdovsky et al. (2017); Lepold et al. (2024)
**Deep Ear Canal**	High mechanical stability due to tight fit; robust during motion and sleep; suitable for long-duration recordings.	Slightly lower amplitude due to reduced contact area; often requires custom-molded earpieces.	Looney et al. (2011); Goverdovsky et al. (2017); Moumane et al. (2024); Nakamura et al. (2019)
**Mixed / Hybrid Placements**	Combines canal and concha or tragus electrodes; improves referencing schemes and robustness.	Increased mechanical complexity; higher inter-subject variability.	Zeydabadinezhad et al. (2024); Musaeus et al. (2022)

### 3.2 Comparative analysis of prior in-ear EEG systems

Table 2 summarizes the key design characteristics of existing in-ear and ear-adjacent EEG systems, including electrode placement, montage configuration, sensing materials, device platforms, and the EEG activities validated in each study. This comparative overview highlights trends across the literature and contextualizes the design choices adopted in recent in-ear EEG developments.

**Table 2:** Comparative analysis of in-ear and ear-adjacent EEG sensor designs reported in literature.

Channels	Electrode location	Montage	Dry/wet	Material	Device	Application	EEG activities	References
Multiple	Ear canal + concha	Benchmarked against scalp EEG	Dry	Ag/AgCl	Custom prototype	Wearable EEG monitoring	Alpha, ASSR	Looney et al. (2012)
2/ear	In-ear canal	Cross-ear electrode configuration (LES–RES, LIE–RIE)	Dry	Silicon + silver ink	NAOX in-ear EEG	Brain activity during wakefulness and sleep	Resting EEG, daytime nap	Moumane et al. (2024)
Multi-channel	Ear-level	Phantom-based ear-EEG validation	Wet	Standard EEG materials	EaR-P Lab	Validation of ear-EEG systems	ASSR, Alpha, MMN, P300, SSVEP	Correia et al. (2024)
8	Ear canal + concha	Right posterior canal as reference	Dry	Ag/AgCl	Aware Hearable System	Ambulatory epilepsy monitoring	Seizure and temporal-lobe activity	Zeydabadinezhad et al. (2024)
4	Concha + outer canal	Ear–scalp, inter-ear, intra-ear montages	Dry	N/A	Nicolet Wireless 64 (256 Hz)	Sleep EEG monitoring	Alpha, K-complexes, spindles, slow-wave sleep	Zibrandtsen et al. (2016)
7	Left + right in-ear	Left–right in-ear dual montage	Dry	SoftPulse ear electrodes	OpenEarable ExG (AD7124-4 ADC)	Cognitive state assessment	Alpha (eyes open/closed)	Lepold et al. (2024)
Multiple	In-ear canal	In-ear referencing	Dry	TPU/Ecoflex	Custom in-ear device	Long-term health monitoring	Alpha, Beta, Delta, Gamma	Zhuo et al. (2025)
6/ear	Ear canal + concha	Bipolar montage (each ear)	Dry	3D-printed custom earpieces	TMSi Mobita EEG	Epileptiform activity in Alzheimer's	Long-term EEG (sleep + wakefulness)	Musaeus et al. (2022)

### 3.3 Material selection and biocompatibility

Material selection in in-ear EEG systems extends far beyond comfort considerations; it fundamentally determines the ESI quality, long-term impedance stability, mechanical conformity, and ultimately the fidelity of neural signals extracted from the highly sensitive ear canal environment. The ear canal is approximately 2.5 cm long with an average diameter of 0.7 cm (Cheng et al., 2015), and its thin, innervated skin makes it one of the most constrained environments for wearable biosensors. As a result, materials used for in-ear EEG electrodes and substrates must exhibit mechanical compliance, biocompatibility, and sufficient electrical conduction without causing irritation or discomfort during extended wear. Contrary to narrow assumptions that electrodes must be constructed using plastic films, recent studies demonstrate the successful use of medical-grade silicone, thermoplastic polyurethane (TPU), Ecoflex foams, soft 3D-printed composites, laminated polymer meshes, and sintered Ag/AgCl coatings, each chosen to balance flexibility, durability, and stability (Zeydabadinezhad et al., 2024; Lepold et al., 2024; Zhuo et al., 2025; Musaeus et al., 2022). Rigid materials such as PET or PI films are generally avoided in the ear canal due to micro-slippage and discomfort.

A key decision concerns the type of electrode material and its associated ESI characteristics. Wet Ag/AgCl electrodes provide low baseline impedance and high-quality contact as explained in Correia et al. (2024), but are unsuitable for multi-hour sessions because the electrolyte gel dries quickly, causing impedance to rise substantially within 30–90 min (Yao and Zhu, 2016; Chi et al., 2010). As a result, modern in-ear EEG systems overwhelmingly adopt dry electrodes. In particular, electrodes fabricated from noble conductive materials such as platinum (Pt), silver (Ag), and gold (Au) are widely used due to their electrochemical stability, biocompatibility, corrosion resistance, and suitability for long-term skin contact. Reported dry-electrode designs include mesh-laminated structures (Norton et al., 2015), conductive silicone electrodes (Moumane et al., 2024), spring-loaded metallic contacts (Chi et al., 2011), and custom-molded Ag/AgCl earplugs (Looney et al., 2012; Goverdovsky et al., 2017). These designs vary dramatically in conductivity, deformation tolerance, surface roughness, and long-term stability—properties that directly impact impedance, SNR, and susceptibility to motion artifacts.

Across studies, a consistent quantitative pattern emerges: sintered Ag/AgCl and Ag/AgCl-plated silver electrodes remain the most reliable dry materials for low-noise in-ear EEG. Looney et al. demonstrated that custom Ag/AgCl canal electrodes maintain impedances of 5–10 kΩ under head movement (Looney et al., 2012), while Goverdovsky et al. (2017). reported comparably low impedance with significantly reduced motion-induced distortion compared to polymer electrodes. Mikkelsen et al. (2015) further showed 30–50% lower noise spectral density for Ag/AgCl electrodes relative to conductive polymer foams. Conversely, polymer-based electrodes (TPU/Ecoflex) exhibit impedances in the 20–50 kΩ range (Zhuo et al., 2025), and spring-loaded metal electrodes frequently exceed 50–70 kΩ unless stabilized mechanically (Chi et al., 2011). These findings collectively indicate that although polymer electrodes offer superior comfort and manufacturability, Ag/AgCl materials provide the highest electrical performance and stability, making them the current gold standard for in-ear EEG sensing.

**Figure 5:**
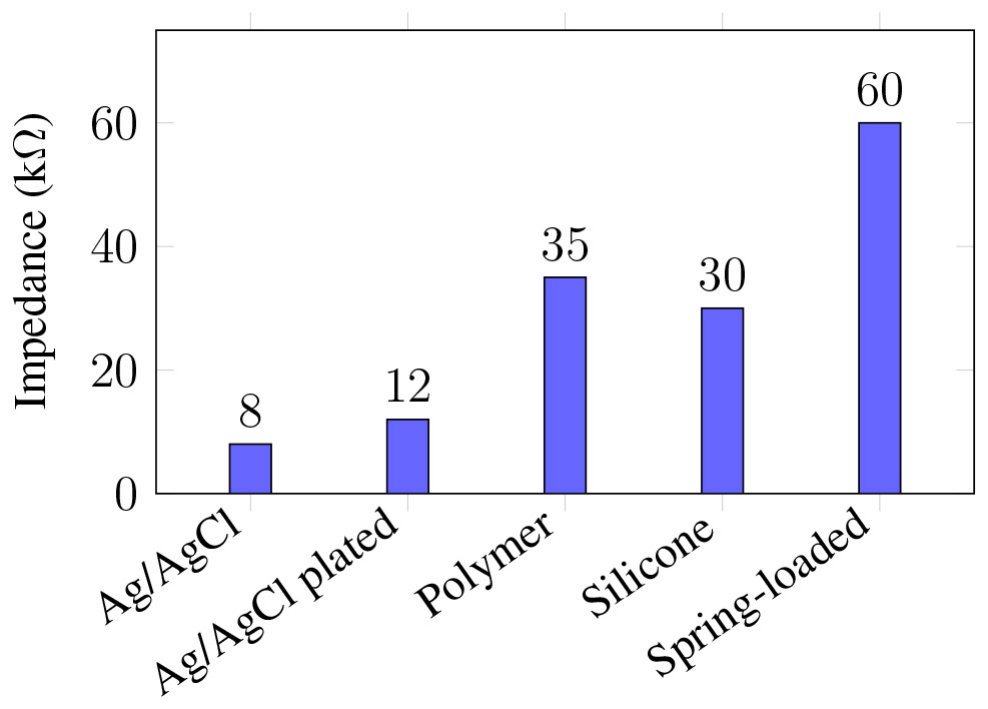
Comparison of typical electro-skin impedance values for commonly used in-ear EEG dry electrode materials based on published ranges (Looney et al., 2012; Goverdovsky et al., 2017; Zhuo et al., 2025; Moumane et al., 2024; Chi et al., 2011).

Table 3 and Figure 5 synthesize the quantitative performance ranges reported across studies, revealing clear stratifications in impedance and noise characteristics across material classes. When combined with the qualitative mechanical and biocompatibility considerations discussed earlier, these data provide a comprehensive foundation for selecting electrode materials in in-ear EEG devices.

**Table 3:** Quantitative comparison of common dry electrode materials used for in-ear EEG systems.

Material type	Typical impedance (kΩ)	Noise/SNR performance	Mechanical properties	Limitations	Representative studies
**Sintered Ag/AgCl**	5–10	Lowest noise; 30%–50% lower noise spectral density vs. polymer electrodes	Rigid core with stable ESI; good long-term contact	Requires molding or custom shaping for ear canal fit	(Looney et al., 2012; Mikkelsen et al., 2015; Goverdovsky et al., 2017)
**Ag/AgCl-plated silver**	8–15	Low motion artifacts; stable drift; high SNR	Durable surface; maintains contact under movement	Slightly higher impedance than sintered Ag/AgCl	(Looney et al., 2012; Goverdovsky et al., 2017)
**Conductive polymer (TPU/ecoflex)**	20–50	Moderate noise; humidity-sensitive; deformation-driven variance	Highly flexible; improved comfort; conforms to canal geometry	Impedance drift with sweat/temperature; pressure sensitive	(Zhuo et al., 2025; Moumane et al., 2024)
**Conductive silicone**	25–40	Higher noise floor; sensitive to bending and deformation	Soft, biocompatible; suitable for long-term wear	Lower conductivity; less stable than metal-based electrodes	(Moumane et al., 2024)
**Spring-loaded metal pins**	50–70	Highest motion artifacts; unstable ESI under movement	Mechanically adaptive; can maintain contact without molding	Uncomfortable for long-term use; highly variable impedance	(Chi et al., 2011)

Several cited studies evaluate and benchmark multiple electrode materials; therefore, a single reference may appear in more than one row to reflect material-specific performance metrics reported in the literature.

The observed differences across materials have direct consequences for the design of in-ear EEG hardware. Ag/AgCl electrodes enable the use of low-gain, low-power AFEs, reducing noise amplification requirements and supporting miniaturized in-ear electronics. In contrast, polymer electrodes with higher and more variable impedance necessitate higher-gain amplifiers, improved shielding, or real-time impedance tracking to maintain SNR during motion. Soft substrates like silicone provide excellent comfort but introduce deformation-induced impedance drift, motivating hybrid composites or mechanical stabilization structures. These trade-offs demonstrate that electrode materials, substrates, and AFEs electronics must be co-designed rather than approached independently.

#### 3.3.1 Remaining research gaps

Despite advances in electrode materials and form factors, several open challenges persist in the design and validation of in-ear EEG electrodes:

- **Lack of standardized evaluation protocols**: Existing studies employ heterogeneous insertion depths, contact pressures, motion paradigms, and impedance reporting practices, limiting meaningful cross-study comparison.
- **Limited long-duration biocompatibility assessment**: The majority of evaluations are restricted to short experimental sessions, with few studies examining continuous wear beyond 24–48 h under real-world conditions.
- **Unmodeled deformation–impedance coupling**: There is no unified framework linking mechanical deformation, ear-canal biomechanics, and time-varying electrode–skin impedance, particularly for soft and polymer-based electrodes.
- **Underexplored ear-canal microclimate effects**: The impact of humidity, temperature, and sweat accumulation on long-term electrode stability and noise characteristics remains insufficiently quantified.
- **Comfort–performance trade-offs**: Systematic investigations correlating mechanical compliance, user comfort, signal-to-noise ratio, and motion robustness are largely absent.

### 3.4 Power source and energy efficiency

The most limiting aspect of wearable technology is the electrical power supply. It restricts autonomy and has a direct impact on the weight and size of devices. Although the use of batteries is still widespread, their cost and impact on the environment is an increasing problem. In general, batteries and energy harvesting from the environment or the human body are the only possible ways to power in-ear devices. In recent times, rechargeable batteries are mostly being used for smart earbuds, i.e., nickel-metal hybrid or lithium-ion rechargeable batteries and a battery charger (Passerini et al., 2000). Rechargeable batteries have limited capacity, require frequent charge, and have a lifespan of less than a year. Wireless charging via an inductive circuit offers some convenience, but it still depends on a single, often bulky device. In general, current solutions remain costly and inconvenient.

Another possibility is to use energy harvesting from the dynamic motion of the ear canal as a source of power. The ear canal is a dynamic environment, and when chewing, smiling, yawning, eating, or speaking, the ear canal wall expands and compresses. Ear canal deformation caused by the activity of the temporomandibular joint (TMJ) is also known as dynamic motion of the ear canal. The TMJ, which is also called the jaw joint, is located near the ear canal, and its slight movement when the mouth opens and closes affects the shape of the ear canal and changes its geometry as explained by the authors in Delnavaz and Voix (2013). In the study Bouchard-Roy et al. (2020), the authors searched for a way to measure the kinetic energy produced by the dynamic movements of the ear canals during meal mastication. For this investigation, a wearable hydraulic ear canal device for power sensing was created to detect the power related to dynamic movements of the ear canal. Six people were assessed for mean power while eating. The findings show that digesting a full meal can easily produce an average power of 26.2 mW in a single ear canal.

By improving the proposed energy harvesters and continuing to reduce the power requirements of electronic devices, it is foreseeable in the near future that dynamic motion of the ear canal could at least partially and eventually fully supply the power needed for hearing devices, electronic hearing protectors or any other in-ear monitor (IEM).

### 3.5 Circuit design considerations for in-ear EEG and multimodal devices

Modern in-ear EEG systems require the co-integration of AFEs, digital processing, wireless communication, and power management within extremely small form factors. The AFE must maintain high input impedance (typically >10 MΩ), low input-referred noise (sub-1 μV_rms_), and strong common-mode rejection (>90 dB) to reliably capture low-amplitude neural signals (< 100 μV, 0.5–40 Hz) under the high impedance variability and motion sensitivity characteristic of ear-EEG recordings (Looney et al., 2011; Goverdovsky et al., 2017; Mikkelsen et al., 2015).

**Figure 6:**
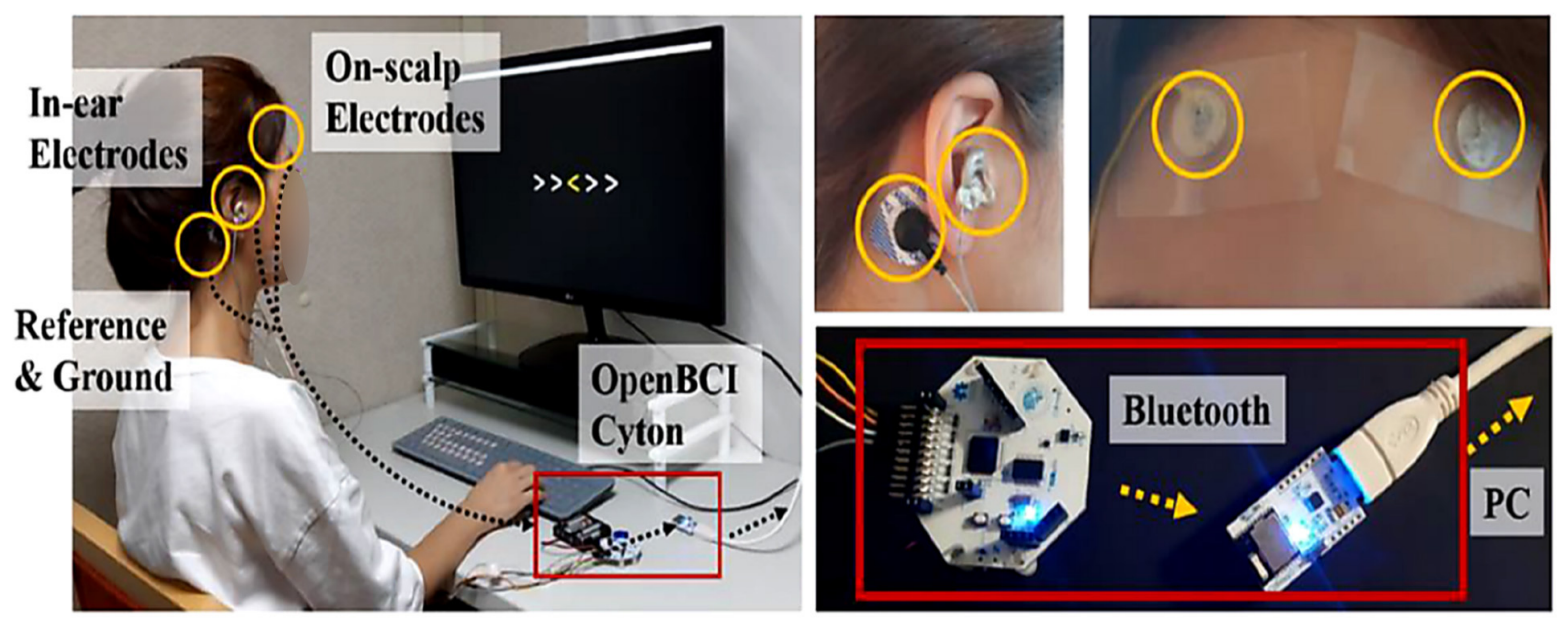
Early in-ear EEG prototype using the OpenBCI Cyton platform introduced in Jeong and Jeong (2020). Modular placement of AFEs, ADCs, and Bluetooth radios illustrates the size limitations of first-generation systems. Reproduced with permission.

Early ear-EEG systems relied on modular development platforms such as OpenBCI (Jeong and Jeong, 2020), where the AFE, digitizer, microcontroller, and wireless telemetry were implemented on multi-cm^2^ PCBs. While these designs enabled rapid prototyping and algorithm development, they exceeded the size, thermal, and ergonomic constraints of the ear canal, as shown in Figure 6. It should be noted that platforms such as OpenBCI Cyton are research-grade systems and not certified medical devices; they are primarily used for rapid prototyping and algorithm development rather than clinical diagnosis or therapeutic decision-making.

**Figure 7:**
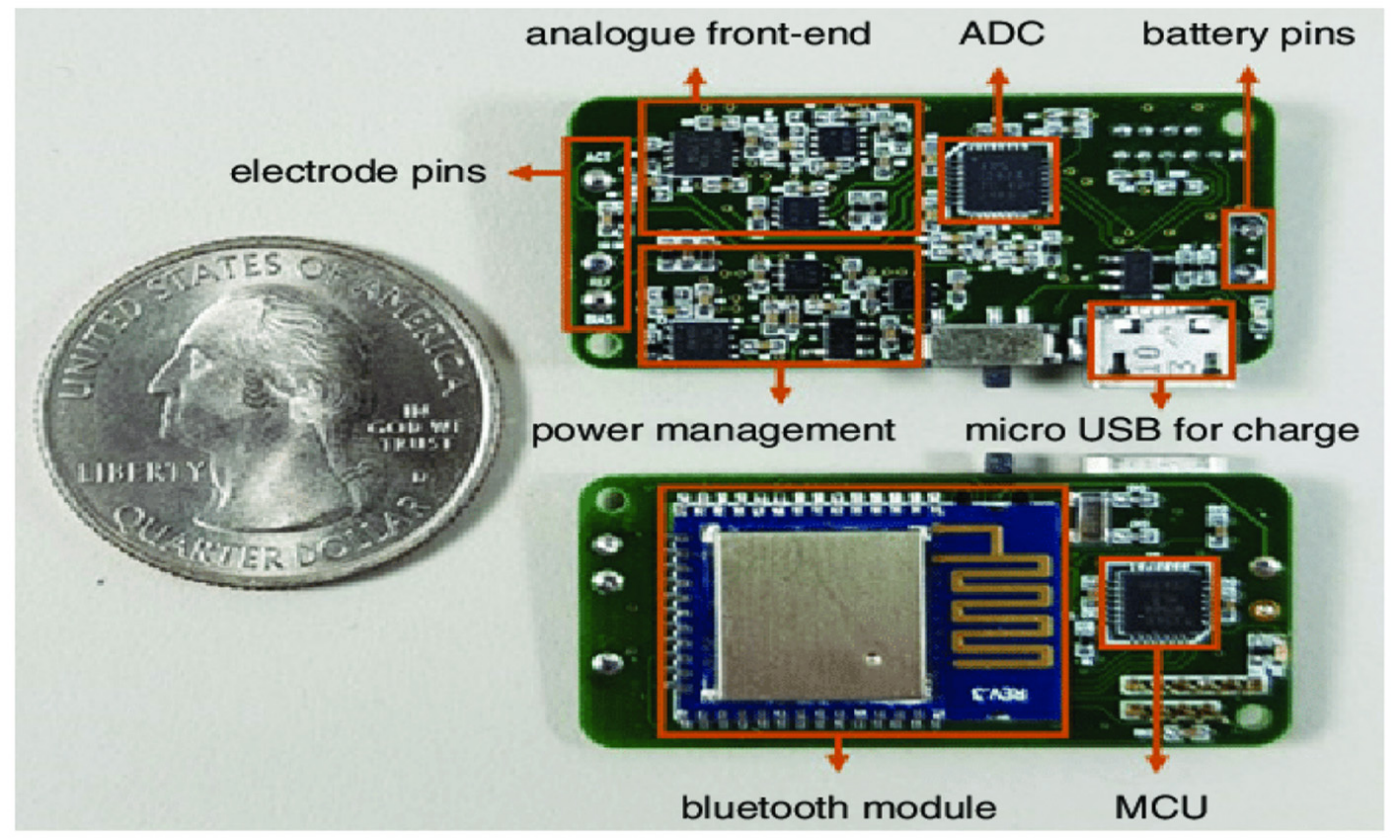
Ahn et al. (2018) proposed miniaturized coin-sized PCB integrating AFE, ADC, MCU, and BLE for in-ear EEG acquisition. Reproduced with permission.

Subsequent work focused on miniaturized PCBs, integrating AFEs, ADCs, microcontrollers, and BLE radios into compact earbud-like footprints (Ahn et al., 2018). These custom designs achieved the size and power constraints required for daily wearable use, but often required trade-offs between wireless bandwidth, sampling rate, and runtime (Figure 7).

A key factor influencing circuit performance is the electrode–electronics interface. Most in-ear EEG devices use passive electrodes directly connected to high-input-impedance AFEs, minimizing in-ear bulk and heat generation but increasing sensitivity to impedance drift and motion artifacts. In contrast, Chi et al. (2011) noted that active electrodes commonly used in scalp EEG place a low-noise buffer amplifier close to the electrode to stabilize the high-impedance interface. Although active electrodes remain underexplored in in-ear devices due to strict power and thermal constraints, recent ultra-low-power analog techniques make their future adoption feasible for improving motion robustness and long-duration stability.

Beyond EEG-only acquisition, multimodal integration has emerged as a major direction. Several recent ear-worn systems combine EEG with photoplethysmography (PPG) and inertial measurement units (IMU), requiring careful electromagnetic interference (EMI) mitigation, synchronized sampling pipelines, and shared power-management strategies to maintain EEG fidelity in the presence of optical and inertial sensors (Frey et al., 2023; Ahn et al., 2018). These multimodal designs offer richer physiological monitoring without increasing the device footprint.

**Figure 8:**
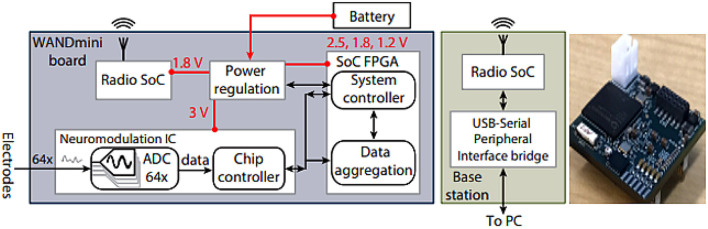
Kaveh et al. (2019) proposed WANDmini closed-loop neural interface platform integrating multichannel AFEs, stimulation drivers, and wireless telemetry. Reproduced with permission.

At the system level, hardware has evolved from simple acquisition modules to fully integrated neural platforms. Kaveh et al. (2019) demonstrated WANDmini (Figure 8), a compact closed-loop neural interface with multichannel AFEs, stimulation drivers, wireless telemetry, and low-latency control, illustrating the trajectory toward neuroscience-grade miniature hardware.

**Figure 9:**
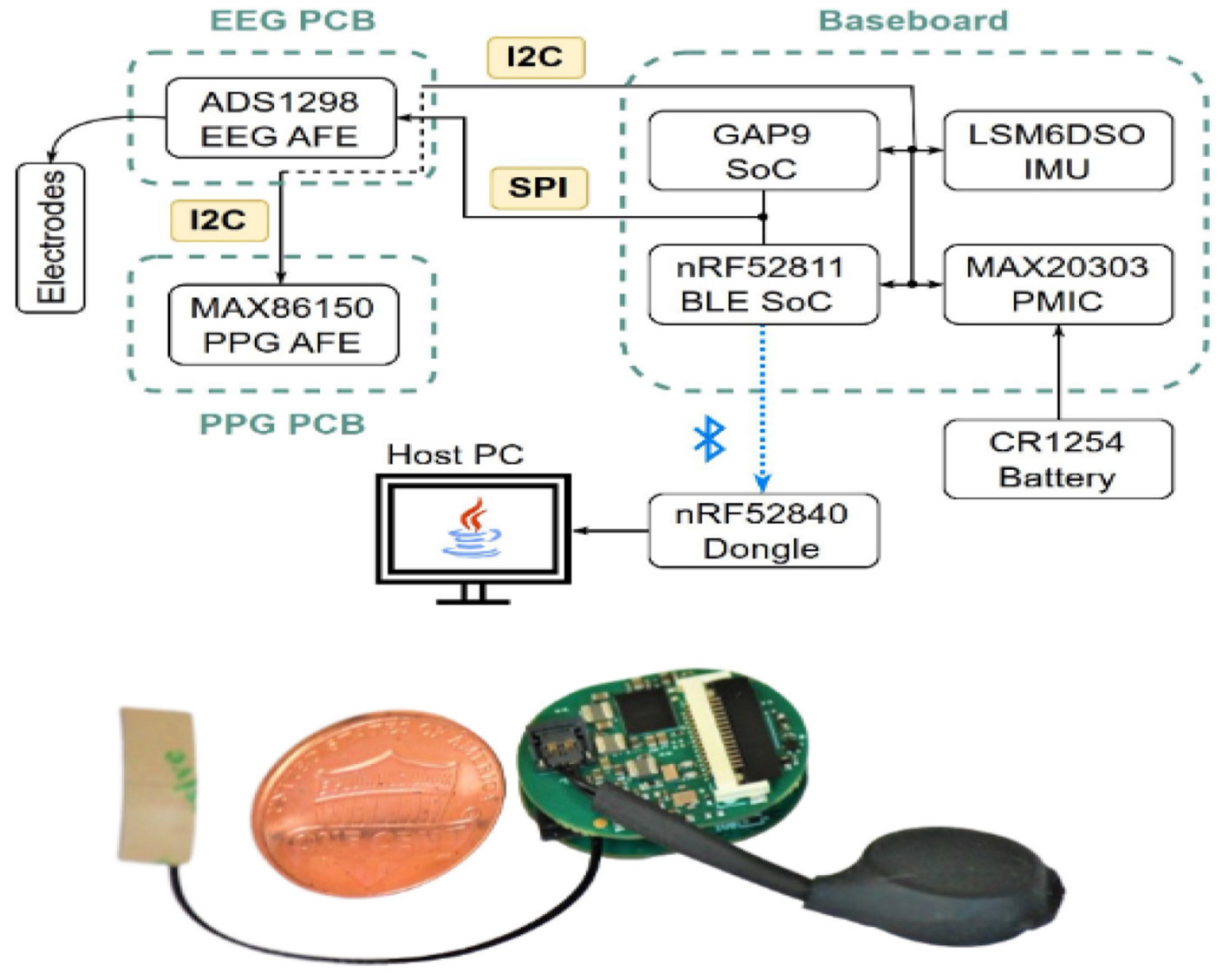
Frey et al. (2023) introduced BioGAP platform based on the GAP9 SoC, integrating EEG, IMU, PPG, and embedded AI accelerators for multimodal ear-worn sensing. Reproduced with permission.

Frey et al. (2023) presented BioGap (Figure 9), integrating low-noise EEG front-ends, IMU and PPG sensing, and embedded AI accelerators within a sub–2 cm^3^ earbud-compatible form factor. Unlike standalone development boards, BioGAP uses GAP9 as a computational core while the platform provides synchronized multimodal sensing, ultra-low-power operation, and on-node inference. This combination positions BioGAP as a state-of-the-art reference for daily-use in-ear neurotechnology.

Performance in these systems is typically evaluated using metrics such as signal quality (e.g., noise floor and signal-to-noise ratio), stability of electrode–skin impedance, ability to capture physiologically relevant EEG rhythms, robustness to motion artifacts, power consumption, and suitability for long-term wearable operation. The progression from modular boards to integrated multimodal ear-worn platforms highlights the need for co-design across circuits, electrodes, and mechanical structures. In practical devices, RF layout, grounding topology, and electrode placement strongly influence noise performance and motion robustness. These insights underscore that successful in-ear EEG hardware requires joint optimization of electronic, mechanical, and anatomical factors rather than circuit design in isolation.

Despite these advances, several hardware aspects remain insufficiently explored in current in-ear EEG systems. These include (i) thermal management strategies tailored to the confined ear canal environment, (ii) lifetime-aware electrode design with self-calibration or impedance-tracking capabilities, (iii) integration of safety and self-diagnostic circuits given proximity to the tympanic membrane, and (iv) combined earmold–circuit co-design to address RF efficiency, comfort, and acoustic sealing. Addressing these gaps will be essential for translating research prototypes into long-term daily-wear neurotechnology that is safe, reliable, and robust across users and activities.

### 3.6 Noise reduction and motion artifact handling

In-ear EEG recordings are highly susceptible to artifacts due to their anatomical proximity to facial muscles, jaw motion, and ocular activity. As expressed in Equation 1, the observed ear-EEG signal is a mixture of neural activity and multiple noise sources, including EOG, EMG, motion-induced impedance changes, and miscellaneous transient artifacts such as blinks, chewing, and speech. These distortions significantly bias spectral features and degrade cognitive-state inference, making robust artifact-mitigation strategies essential for reliable interpretation of in-ear EEG.


$$\begin{array}{l} \begin{array}{c} EEG_{\text{observed}}\left(t\right)=EEG_{\text{source}}\left(t\right)\\ +\;EOG\left(t\right)+EMG\left(t\right)+Artifacts \end{array} \end{array}$$ (1)

**Figure 10:**
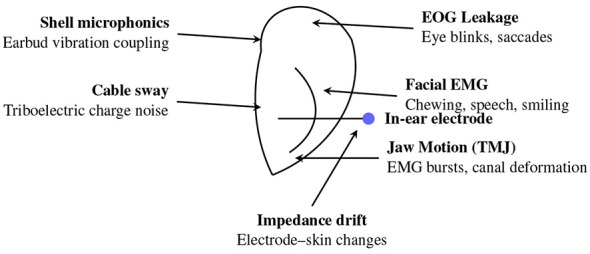
Schematic illustration of major artifact sources in in-ear EEG recordings. Jaw motion deforms the ear canal, facial EMG and EOG leak into the signal, while earbud-shell vibration, cable sway, and electrode–skin impedance drift introduce additional noise and instability.

Figure 10 illustrates the primary artifact sources affecting in-ear EEG recordings. Because the electrode resides within the ear canal, jaw motion from temporomandibular joint (TMJ) activity produces mechanical deformation, generating large EMG bursts and electrode displacement. Facial muscles inject broadband EMG noise during speech, chewing, or smiling, while eye blinks and saccades introduce EOG leakage that propagates through the head volume. Additional disturbances arise from earbud-shell vibration (microphonic coupling), cable-sway triboelectric effects, and electrode–skin impedance drift near the canal wall. Collectively, these sources shape a noise profile that is distinct from scalp EEG and motivate specialized artifact-suppression strategies for ear-EEG.

**Figure 11:**
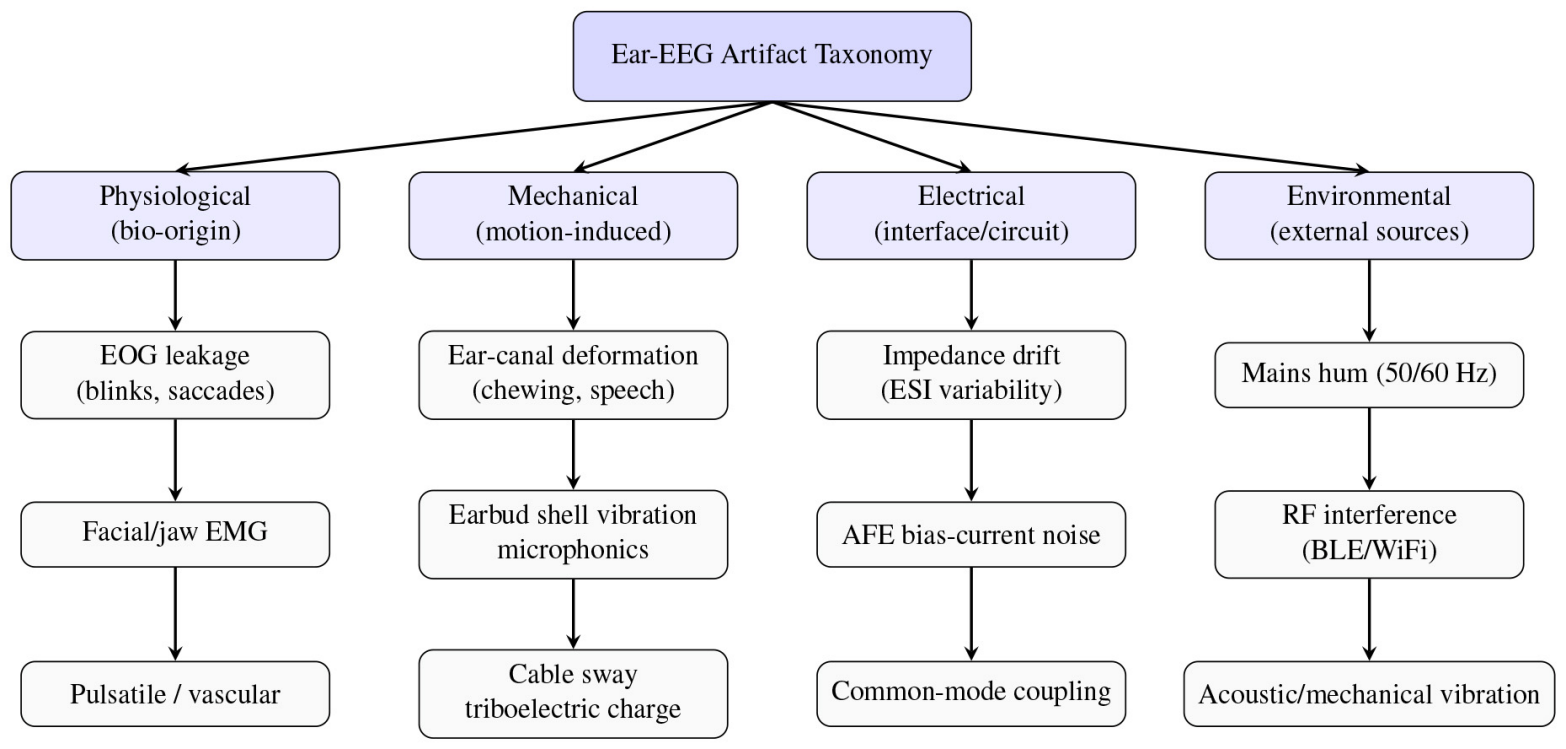
Taxonomy of artifact types affecting ear-EEG systems. Artifacts are grouped into physiological (EOG/EMG/pulsatile), mechanical (ear-canal deformation, microphonics, and triboelectric noise), electrical (impedance drift, bias-current noise, and common-mode coupling), and environmental (mains hum, RF, and acoustic vibration) categories.

Beyond identifying individual artifact generators, it is useful to organize these disturbances into broader categories based on their physiological, mechanical, electrical, or environmental origin. Figure 11 summarizes a high-level taxonomy of artifact classes that commonly affect ear-EEG measurements.

This taxonomy highlights the diversity of noise mechanisms in in-ear EEG systems and underscores the need for tailored artifact-mitigation strategies. Traditional signal-processing techniques such as adaptive filtering (Chandrakar and Kowar, 2012; Correa et al., 2007) and blind source separation methods (ICA/BSS) (Potter et al., 2002; Chawla, 2011; Snyder et al., 2015) remain widely applied for EEG artifact suppression. Adaptive filtering has been shown to reduce various types of motion contamination in mobile EEG and ECG recordings, whereas ICA-based methods can substantially improve SNR and classification accuracy but typically require multichannel data and incur high computational cost (Winkler et al., 2015). These requirements make ICA-based approaches poorly suited for ear-EEG systems, which commonly employ only 1–2 electrodes and operate under strict power constraints.

Goverdovsky et al. (2017) introduced IMU-assisted artifact regression in ear-EEG, where inertial or mechanical sensors embedded within the earpiece serve as motion references to suppress jaw-motion and canal-deformation artifacts. Lightweight methods such as template subtraction and spatial filtering also remain attractive because they impose low computational load and do not require extensive channel counts.

Beyond classical techniques, modern approaches leverage machine learning and deep learning to enhance artifact removal. For instance, IC-U-Net introduced by Chuang et al. (2022), which is a U-Net-based denoising autoencoder developed for mobile EEG, can automatically suppress blinks, muscle activity, and line noise. However, these models face challenges including high computational requirements, dependence on large labeled datasets, potential distortion of neural information, and difficulty generalizing to unseen artifact types—limitations that restrict their use in resource-constrained wearable ear-EEG devices.

To address the need for a systematic comparison, Table 4 summarizes major artifact-removal methods, highlighting their operational principles, computational demands, channel requirements, and suitability for real-world in-ear systems.

**Table 4:** Advanced systematic comparison of artifact-removal methods applied in ear-EEG and wearable EEG systems.

Method category	Principle of operation	Strengths	Major limitations	Computational load	Channel requirement	Suitability
**Adaptive filtering**	Real-time adjustment using reference channels (IMU/EOG/EMG).	Low power; real-time; effective for motion artifacts.	Requires high-quality reference signal.	Very Low	1 EEG + 1 reference	High
**BSS/ICA/PCA**	Statistical decomposition into independent components.	Excellent artifact suppression.	Needs multi-channel signals; high compute.	High	≥4 channels	Low
**Spatial filtering**	Uses electrode geometry to enhance neural sources.	Low compute; interpretable.	Limited by ear-canal electrode spacing.	Low	≥2 channels	Moderate
**Reference sensor fusion**	Regression using IMU or optical motion sensors.	Strong performance for jaw/talking/chewing artifacts.	Requires additional sensors; calibration.	Moderate	1 EEG + IMU	High
**Template subtraction**	Learns and subtracts artifact waveforms.	Very efficient; channel-light.	Fails for non-repetitive EMG artifacts.	Very Low	1–2 channels	Moderate
**Classical ML**	Feature-based artifact detection and filtering.	Lightweight; runs on embedded hardware.	Needs handcrafted features; limited generalization.	Low–Moderate	1–2 channels	High
**Deep learning**	CNN/RNN/U-Net models learn nonlinear artifact patterns.	State-of-the-art suppression.	High computational cost; risk of neural signal attenuation.	High–very high	1 channel acceptable	Moderate
**Denoising autoencoders**	Reconstruct clean EEG without labels.	Robust to complex artifacts.	Still heavy for embedded wearables.	High	1 channel	Moderate
**Hybrid models**	Combines filtering + sensor fusion + light ML.	Best trade-off for wearables.	Still under development/integration stage.	Moderate	1–2 channels	High

Following these classical and AI-assisted approaches, recent work also highlights the importance of monitoring ESI to maintain signal integrity. For example, Sheeraz et al. (2024) demonstrated an ear-wearable EEG system for early Autism Spectrum Disorder detection that integrates real-time ESI monitoring within the analog front end, enabling the detection of impedance drift or degraded electrode contact and prompting corrective action. Although embedding such functionality in a miniaturized wearable AFE remains challenging, the combination of classical filtering, sensor fusion, AI-based denoising, and real-time impedance monitoring represents a comprehensive toolset for improving the robustness of in-ear EEG in motion-rich, everyday environments.

Despite this progress, systematic evaluations of artifact-mitigation pipelines tailored specifically to low-channel, in-ear EEG—using common benchmark datasets and realistic motion conditions are still largely missing and represent an important avenue for future work.

### 3.7 Verification and clinical validation of in-ear EEG systems

Clinical validation of in-ear EEG systems requires demonstrating that signals acquired from the ear canal reliably preserve electrophysiological features measured using established scalp-EEG platforms. Unlike exploratory feasibility studies, clinical-style validation emphasizes reproducibility, quantitative agreement, and functional relevance under both controlled and ambulatory conditions. Accordingly, prior work has evaluated waveform morphology, spectral power distributions, event-related responses, and sleep-related biomarkers to assess whether in-ear EEG can support medical or preclinical use cases (Looney et al., 2012; Kidmose et al., 2013a; Guermandi et al., 2022)

**Figure 12:**
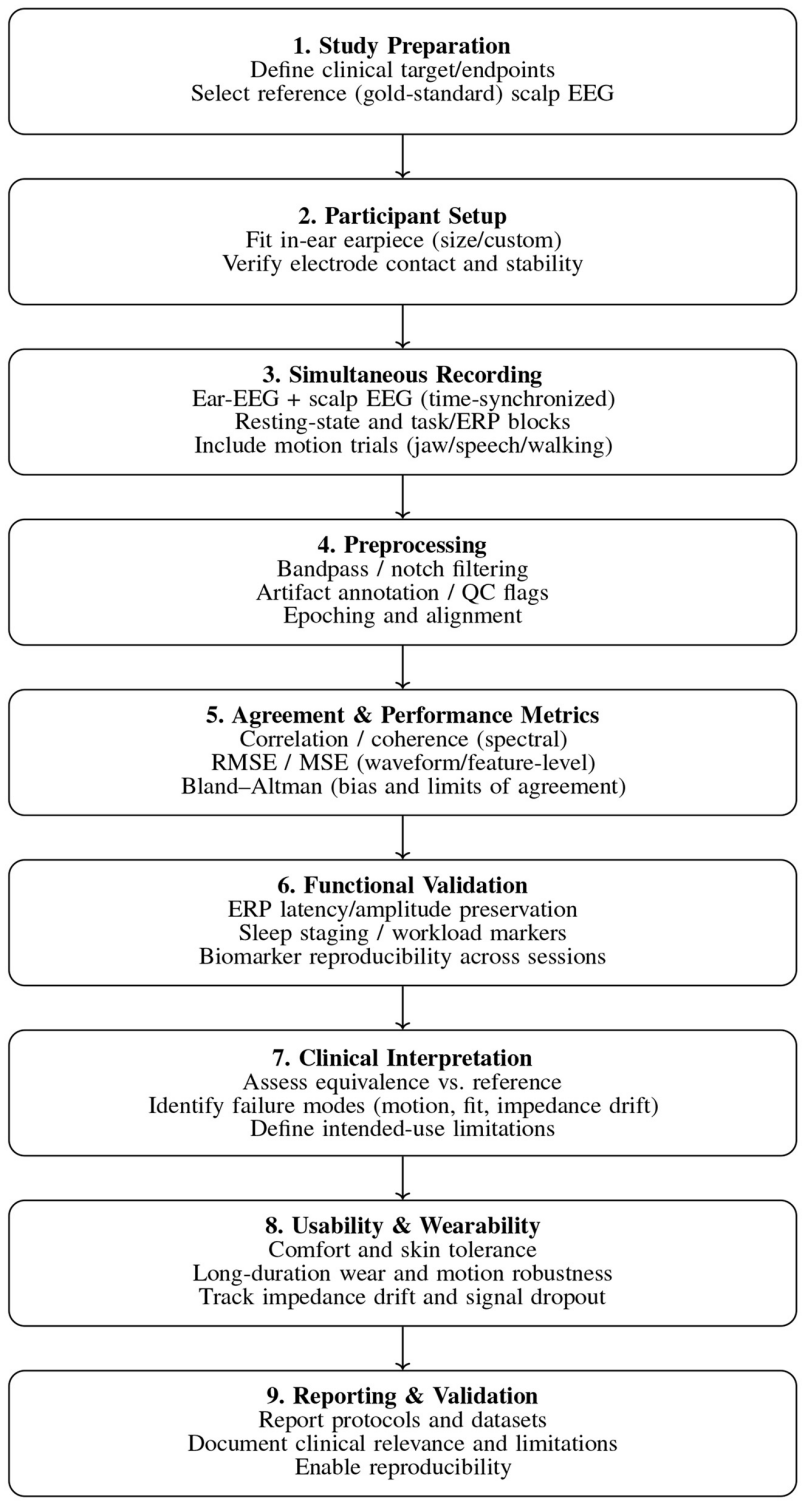
Clinical-style validation workflow for in-ear EEG systems against a reference scalp EEG.

In the literature, validation protocols typically involve simultaneous ear-scalp acquisition followed by quantitative similarity analysis using correlation, coherence, root-mean-square error, and Bland–Altman statistics (Figure 12), with functional validation assessing whether physiologically meaningful markers—such as spectral rhythms, event-related potentials, or sleep-stage features—are preserved across modalities (Kidmose et al., 2013a; Guermandi et al., 2022; Zibrandtsen et al., 2017).

Across multiple cohorts and experimental paradigms, prior studies report that in-ear EEG demonstrates strong agreement with scalp EEG within limits commonly reported in the literature. Resting-state and sleep studies indicate that spectral power estimates and sleep-stage classifications derived from ear-EEG are broadly consistent with polysomnography, achieving substantial agreement in manual and automated staging frameworks (Mikkelsen et al., 2021; Stochholm et al., 2016). Event-related potential investigations further suggest that ERP latencies and amplitudes measured in the ear canal deviate on the order of tens of milliseconds and microvolts relative to scalp recordings, remaining within ranges compatible with cognitive and neurophysiological assessment (Kidmose et al., 2013a; Looney et al., 2012).

In addition to signal-level agreement, motion robustness and long-term wearability are central to the clinical validation of ear-centered EEG systems. To contextualize these validation outcomes, Table 5 contrasts in-ear EEG wearables with traditional clinical EEG systems across form factor, signal quality, power consumption, and usability. This comparison highlights why validation criteria for ear-EEG must differ from those applied to conventional scalp-based systems.

**Table 5:** Comparison of in-ear wearable devices with traditional clinical systems.

Feature	In-ear wearable device	Traditional EEG/ECG systems	Challenges & considerations
**Form factor**	Small, earbud-like	Bulky, wired setups	Limited space for hardware
**Power consumption**	Ultra-low (< 2 mW)	High (>50 mW)	Balancing efficiency & performance
**Signal quality**	Moderate (motion-sensitive)	High (gold-standard accuracy)	Motion artifacts due to jaw/head movement
**Data transmission**	Wireless (BLE, Wi-Fi)	Wired (USB, Ethernet)	Power vs. transmission trade-off
**User comfort & Fit**	High (lightweight, discreet)	Low (head caps, adhesive electrodes)	Stability without causing pressure or discomfort
**Electrode type**	Dry electrodes, capacitive	Wet electrodes (gel-based)	Dry electrodes have higher impedance, lower signal quality
**Battery life**	Extended (>600 h on Zinc-Air)	Limited (few hours on Li-ion)	Battery size vs. usability trade-off
**Portability**	Very high (wearable, daily use)	Low (requires setup)	Compact design without signal degradation

Ear-canal–anchored electrodes offer improved mechanical stability for ambulatory recordings compared with externally mounted sensors, although speech- and chewing-related artifacts remain a persistent limitation (Zibrandtsen et al., 2016; Musaeus et al., 2022). Recent validation studies increasingly emphasize impedance stability, user comfort during extended wear, and signal continuity under daily-life conditions. Long-term outpatient evaluations and wireless ear-EEG systems have demonstrated the feasibility of sustained monitoring while preserving clinically meaningful neural features, underscoring the potential of in-ear EEG for ambulatory neurophysiology, sleep assessment (Kiarashi et al., 2025), and unobtrusive cognitive monitoring (Pazuelo, 2024; Lepold et al., 2024; Guermandi et al., 2022; Paul et al., 2023).

### 3.8 Spatial sensitivity and source coverage in in-ear EEG

Although device validation often focuses on metrics such as SNR or ERP detection, an equally critical yet underexplored dimension is spatial sensitivity, the extent to which in-ear electrodes can capture signals from different regions of the brain. This section provides an overview of modeling studies and empirical validations related to source coverage in in-ear EEG configurations.

Due to anatomical constraints, in-ear systems are inherently limited in spatial coverage compared to full-cap scalp EEG, often being more sensitive to signals originating from the temporal lobes. For example, authors in Meiser et al. (2020); Bleichner and Debener (2017) demonstrated through realistic forward modeling that cEEGrid electrodes primarily capture activity from temporal sources, while sensitivity decreases sharply for deeper or more distant regions. Similarly, in Yarici et al. (2023) extended this modeling to show that although non-temporal sensitivity is limited, fidelity remains for certain eye-related signals, and in Yarici et al. (2023) the authors quantified these losses across cortical areas in decibel terms. Despite these findings, most validation studies continue to rely on basic signal metrics such as SNR or ERP amplitude, offering limited insight into the spatial resolution and directional sensitivity of different in-ear configurations. We propose that future work should integrate source localization techniques, such as lead field mapping, minimum norm estimation (MNE), and beamforming, alongside empirical task-based validation (e.g., auditory or cognitive paradigms) to assess regional coverage more systematically. Such approaches will help optimize electrode placement for specific applications, guide hardware design, and ultimately improve the interpretability of in-ear EEG data in real-world use. Furthermore, the development of open-access spatial sensitivity maps and standard protocols to benchmark coverage across various user anatomies would be valuable steps toward establishing spatial evaluation as a standard component of in-ear EEG validation.

### 3.9 Beyond linear metrics: nonlinear approaches to signal similarity

Conventional evaluations of EEG signals, particularly when contrasting in-ear and scalp recordings, depend mainly on linear metrics such as Pearson's correlation, root mean square (RMS) amplitude, power spectral density (PSD) and SNR. Despite their common usage, these approaches assume straightforward linear connections and do not consider the brain's inherently nonlinear and dynamic nature. This constraint is especially applicable to EEG in the ear, where signals frequently show low amplitude and might not show robust linear correlations with scalp recordings, even though they record significant neural activity. Nonlinear approaches can uncover deeper and intricate relationships and are particularly useful in areas such as emotion detection, cognitive load evaluation, and sleep microstate examination. Multiple studies highlight this necessity: Cukic et al. (2018) showed that nonlinear characteristics such as Higuchi Fractal Dimension and Sample Entropy achieved over 90% classification accuracy in differentiating depression patients from healthy individuals. In a similar study, Pu et al. (2016) monitored EEG alterations after spinal injury in rats employing sample entropy, detrended fluctuation analysis, and Kolmogorov complexity, demonstrating a significant decline in signal complexity after injury, accompanied by gradual recovery over time. In a foundational review, Stam (2005) recommended incorporating nonlinear EEG and MEG metrics such as Lyapunov exponents, correlation dimension, and different entropies, highlighting their ability to represent the complexity of neural dynamics beyond conventional measures. Additionally, nonlinear functional connectivity methods such as cross-sample entropy have been shown to be effective in emotion recognition tasks, further showcasing their usefulness across various fields. In the future, in-ear EEG studies would benefit from incorporating these nonlinear techniques in addition to linear approaches, allowing for a stronger and more accurate physiological understanding of neural signals (Garćıa-Mart́ınez et al., 2021). Future studies should incorporate these metrics into standard evaluation toolkits, compare them with traditional methods, and investigate their integration into machine learning models for practical cognitive and affective uses.

## 4 Multimodal sensing and stimulation in ear-EEG systems

In-ear EEG systems increasingly integrate additional sensing and stimulation modalities to improve signal quality, contextual awareness, and functional utility. Unlike general-purpose earables, the focus here is on multimodal configurations in which auxiliary sensors or stimulation pathways are co-located with ear-EEG to enhance neural signal interpretation, artifact characterization, or closed-loop neurophysiological interaction. The anatomical proximity of the ear canal to vascular, muscular, and neural structures enables simultaneous acquisition of EEG together with complementary biosignals such as photoplethysmography (PPG), electro-oculography (EOG), motion, and acoustic signals, while also supporting localized stimulation paradigms relevant to EEG-based applications.

### 4.1 Multimodal sensing integrated with ear-EEG

Beyond standalone neural recording, ear-centered EEG systems increasingly incorporate complementary sensing modalities (Röddiger et al., 2022) to improve robustness, contextual interpretation, and clinical relevance. The confined anatomy of the ear canal enables stable co-location of electrophysiological, optical, thermal, biochemical, and motion sensors, allowing multimodal data acquisition without substantially increasing device footprint. Importantly, in this context, additional modalities are not treated as independent measurements, but rather as supporting channels that enhance the interpretability, artifact rejection, or functional assessment of ear-EEG.

Optical sensing, particularly photoplethysmography (PPG) and pulse oximetry, has emerged as the most mature complement to ear-EEG. The ear's dense and relatively motion-stable vasculature enables reliable estimation of heart rate, heart-rate variability, and oxygen saturation, even under ambulatory conditions where peripheral sites such as the finger are susceptible to vasoconstriction and motion artifacts (Passler et al., 2019; Davies et al., 2020, 2022). When combined with ear-EEG, PPG-derived cardiac markers provide valuable physiological context for interpreting neural signatures of stress, arousal, sleep, and autonomic regulation (Tian et al., 2023; Goverdovsky et al., 2017). Hybrid approaches that fuse PPG with EEG-derived features have further been explored to support applications such as blood-pressure estimation and fatigue monitoring, although reliable ECG acquisition within the ear remains challenging and algorithmically demanding (Bui et al., 2019; Żyliński et al., 2024).

Thermal sensing represents another modality that complements ear-EEG by capturing slow-varying physiological states. Infrared tympanic thermometry integrated into earables enables continuous approximation of core body temperature, which is relevant for circadian rhythm analysis, infection monitoring, and sleep studies (Ota et al., 2017; Schilk et al., 2022). In multimodal configurations, temperature trends can contextualize EEG-derived sleep stages or vigilance markers, although measurement accuracy remains sensitive to ear-canal geometry, fit, and user motion (Chaglla E et al., 2018).

Biochemical sensing within the ear has recently attracted attention as a longer-term direction for multimodal neurophysiological monitoring. Sweat-based electrochemical sensors have demonstrated the feasibility of measuring lactate, glucose, cortisol, pH, and electrolyte concentrations using flexible or miniaturized electrodes positioned near the ear canal (Gil et al., 2019; Xu et al., 2023). While these biomarkers are not direct surrogates of neural activity, their integration with ear-EEG opens opportunities for correlating metabolic or stress-related states with changes in neural rhythms, particularly in applications such as fatigue monitoring, rehabilitation, and chronic disease management. However, substantial challenges remain related to inter-individual variability in sweat production, environmental sensitivity, and long-term sensor stability (Heikenfeld et al., 2018; Sim et al., 2022).

Electro-oculography (EOG) constitutes a uniquely synergistic modality when combined with ear-EEG. Ear-centered EOG exploits the corneo-retinal dipole using electrodes placed in or around the ear canal, enabling detection of blinks, saccades, and slow eye movements with lower amplitude but sufficient reliability for fatigue and workload monitoring (Favre-Félix et al., 2017; Skoglund et al., 2022). Critically, EOG signals acquired at the ear can be leveraged to identify and suppress ocular artifacts in ear-EEG, improving neural signal quality without requiring additional facial electrodes. Despite susceptibility to cross-talk and motion-related interference, ear-EOG remains one of the most functionally relevant adjuncts to ear-EEG in real-world settings as mentioned by An et al. (2025).

Finally, mechano-acoustic and inertial sensing provide indirect yet valuable support for ear-EEG interpretation under ambulatory conditions. Sensors capturing ear-canal deformation, vibration, and head kinematics have been used to identify gait patterns, chewing, speech, and gross motion, enabling detection of Parkinsonian gait or fall risk while simultaneously monitoring neural activity (Oishi et al., 2021; Atallah et al., 2014). When co-recorded with ear-EEG, these signals offer reference channels for motion-artifact characterization and rejection, although they are inherently sensitive to environmental noise and non-task-related movements (Burgos et al., 2020; Moschina et al., 2023).

Table 6 summarizes the primary sensing modalities that have been integrated with ear-EEG, highlighting their associated biomarkers, representative applications, and key limitations. Collectively, these studies indicate that multimodal sensing is most effective when additional channels are explicitly leveraged to stabilize, contextualize, or enhance ear-EEG rather than functioning as independent measurements. This paradigm positions ear-EEG not merely as a compact alternative to scalp EEG, but as a central node within a tightly coupled multimodal neurophysiological sensing framework.

**Table 6:** Representative studies integrating ear-EEG with additional sensing or stimulation modalities.

References	Integrated modalities	Target application	Key contribution/benefit	Limitations reported
Favre-Félix et al. (2017)	Ear-EEG + ear-centered EOG	Ocular artifact detection and eye-movement monitoring	Demonstrated reliable detection of blinks and saccades using ear-centered electrodes, enabling EOG-assisted artifact identification in ear-EEG recordings.	EEG/EOG cross-talk necessitates careful electrode placement and signal separation.
Skoglund et al. (2022)	Ear-EEG + ear-centered EOG	Drowsiness and vigilance assessment	Showed improved detection of ocular artifacts and vigilance-related features in ear-EEG under realistic conditions.	Reduced amplitude compared to periocular EOG; sensitivity depends on ear anatomy and fit.
Debener et al. (2015)	Ear-EEG + motion sensors	Ambulatory EEG during daily activities	Demonstrated feasibility of mobile ear-centered EEG under movement conditions.	Motion artifacts remain challenging without explicit inertial correction.
Bleichner and Debener, 2017	Ear-EEG + motion-aware evaluation	Unobtrusive EEG acquisition	Established practical limits of ear-EEG under real-world motion.	Limited spatial coverage and sensitivity to head movement.
Kappel et al. (2019)	Ear-EEG + motion robustness analysis	Dry-electrode ear-EEG validation	Reported improved stability for ambulatory EEG recordings.	Lower signal amplitude compared to wet scalp EEG.
Guermandi et al. (2022)	Ear-EEG + IMU + wireless streaming	Auditory evoked responses; mobile EEG	Demonstrated fully untethered ear-EEG with motion context; IMU used to identify movement-related artifacts.	Limited channel count; sensitivity mainly to temporal sources.
Zibrandtsen et al. (2016)	Ear-EEG + clinical monitoring	Epileptiform discharge detection	Showed feasibility of prolonged outpatient EEG monitoring using ear-centered electrodes.	Lower spatial resolution than scalp EEG.
Paul et al. (2023)	Ear-EEG + surface EEG + wearable DAQs	Multimodal neurophysiology benchmarking	Direct comparison between ear-EEG and scalp EEG during naturalistic tasks.	Bulkier setup than consumer earables.
Lepold et al. (2024)	Ear-EEG + IMU + microphone + PPG	Open-source multimodal earable platform	Introduced modular hardware enabling synchronized ear-EEG with motion and acoustic sensing.	Performance depends on user-specific fit.
An et al. (2025)	Ear-EEG + ear-EOG	Drowsiness and fatigue monitoring	Reported improved ocular artifact identification and vigilance assessment using ear-EOG.	Cross-talk requires advanced signal separation.
Pazuelo (2024)	Ear-EEG + long-term outpatient monitoring	Clinical feasibility evaluation	Evaluated signal stability and usability of ear-EEG during extended daily-life use.	Limited subject cohort.

Multimodal ear-EEG systems are most effective when auxiliary sensors are leveraged as contextual and corrective channels rather than independent diagnostic modalities. Optical, inertial, and EOG signals primarily enhance ear-EEG by (i) stabilizing neural feature extraction under motion, (ii) disambiguating autonomic versus cortical contributions to low-frequency rhythms, and (iii) enabling artifact-aware feature selection. Architectures that treat multimodal data as parallel outputs—rather than EEG-conditioned inputs—often incur redundancy, increased power consumption, and reduced interpretability.

Despite promising demonstrations, several fundamental questions remain unresolved. There is currently no consensus on (i) how multimodal features should be temporally aligned with ear-EEG given modality-dependent latencies, (ii) which auxiliary signals most reliably generalize across users and daily-life contexts, and (iii) how multimodal fusion impacts long-term calibration drift and electrode–skin impedance variability in ear-centered EEG. Addressing these challenges is critical for translating multimodal ear-EEG from proof-of-concept prototypes into clinically robust and scalable platforms.

### 4.2 Stimulation integrated with ear-EEG: opportunities and open challenges

In-ear stimulation extends ear-EEG systems from passive monitoring platforms toward interactive neurophysiological interfaces. In contrast to standalone neuromodulation devices, stimulation in ear-EEG systems is uniquely positioned to operate in close anatomical proximity to both peripheral neural pathways and EEG sensing electrodes, enabling bidirectional sensing–stimulation paradigms within a compact form factor. However, despite growing interest, the integration of stimulation with ear-EEG remains fragmented, with most studies treating stimulation and EEG recording as loosely coupled or sequential processes rather than as tightly co-designed system components.

**Acoustic stimulation** is the most mature modality integrated with ear-EEG, leveraging miniature speakers or bone-conduction transducers already present in hearing devices. Acoustic stimuli have been widely used to elicit auditory steady-state responses (ASSRs), auditory brainstem responses (ABRs), and sleep-related oscillatory entrainment, making them particularly compatible with EEG-based assessment of auditory processing, vigilance, and sleep (Henry, 2022; Montazeri et al., 2023). In ear-EEG systems, acoustic stimulation primarily serves as a controlled input for probing neural responsiveness rather than as a therapeutic intervention. A largely unexplored challenge is the interaction between acoustic actuation and mechanical microphonics at in-ear EEG electrodes, which can contaminate low-frequency EEG bands and confound interpretation if not explicitly modeled or compensated.

**Electrical stimulation**, particularly transcutaneous auricular vagus nerve stimulation (taVNS), represents the most biologically targeted form of in-ear neuromodulation. Electrodes placed on the cymba conchae or tragus can activate the auricular branch of the vagus nerve, influencing central autonomic and neuromodulatory circuits (Frangos et al., 2015; Yakunina et al., 2017). While several studies have demonstrated changes in EEG rhythms and functional connectivity during or after taVNS, current implementations rarely exploit ear-EEG to close the loop in real time. Critically, there is no consensus on how stimulation artifacts should be separated from neural activity in ear-EEG recordings, nor on which EEG features are most reliable for adaptive taVNS control. Moreover, stimulation parameter selection (current amplitude, pulse width, and duty cycle) remains largely heuristic and task-specific, limiting reproducibility across studies.

**Vibrotactile stimulation** has been explored primarily for haptic feedback, balance assistance, and human–computer interaction. Although not directly targeting neural tissue, vibrotactile actuation induces somatosensory and vestibular responses that are observable in EEG, particularly in sensorimotor and parietal regions. In ear-EEG systems, vibrotactile stimulation introduces a unique challenge: mechanical coupling between actuators and electrodes can generate motion artifacts that overlap spectrally with neural signals of interest. To date, few studies have quantitatively characterized this interaction or developed co-design strategies that jointly optimize stimulation delivery and EEG signal integrity.

Beyond individual modalities, a major unmet need lies in the development of closed-loop stimulation architectures explicitly grounded in ear-EEG. While conceptual frameworks for closed-loop neuromodulation are well established, their translation to ear-centered systems raises unresolved questions regarding latency constraints, feature robustness under motion, and long-term calibration drift. In particular, it remains unclear (i) which ear-EEG biomarkers are sufficiently stable for continuous stimulation control, (ii) how stimulation-induced changes in electrode impedance affect concurrent EEG acquisition, and (iii) how multi-modal stimulation strategies can be coordinated without violating power and safety constraints.

Overall, stimulation-integrated ear-EEG remains an underexplored design space. Progress in this area will require moving beyond proof-of-concept demonstrations toward system-level co-design, where electrode placement, stimulation geometry, signal processing, and control policies are jointly optimized. Addressing these challenges is essential for transforming ear-EEG from a monitoring technology into a clinically actionable platform for adaptive neurophysiological intervention.

### 4.3 Integration of sensing and stimulation

The integration of sensing and stimulation in ear-centered EEG systems enables a class of closed-loop neurophysiological interfaces that are fundamentally different from conventional open-loop earables and scalp-based neuromodulation platforms. Owing to the tight anatomical co-location of electrodes, actuators, and biosignal sources within the ear canal, ear-EEG systems introduce unique opportunities—but also unique constraints—for real-time, adaptive stimulation driven by neural state estimation.

Unlike conventional closed-loop neurostimulation systems, ear-EEG–based architectures must contend with strong bidirectional coupling between sensing and actuation. Mechanical vibrations, electrical stimulation currents, and acoustic actuation can directly perturb in-ear electrodes, producing artifacts that overlap spectrally with low-frequency EEG rhythms. At the same time, stimulation can alter electrode–skin impedance and local tissue properties, introducing non-stationarities that evolve over time. These effects necessitate joint co-design of electrode geometry, stimulation drivers, artifact-aware signal processing, and control policies—an aspect largely absent from existing closed-loop frameworks. As a result, many reported systems remain confined to short-duration laboratory demonstrations, with limited evidence of robustness under prolonged wear, user motion, or inter-session variability. In particular, the absence of standardized strategies for handling stimulation-induced non-stationarities, impedance drift, and latency-aware control severely limits reproducibility and clinical scalability. Addressing these system-level challenges is essential for transitioning ear-EEG closed-loop architectures from experimental prototypes to reliable, long-term neurotherapeutic platforms.

**Figure 13:**
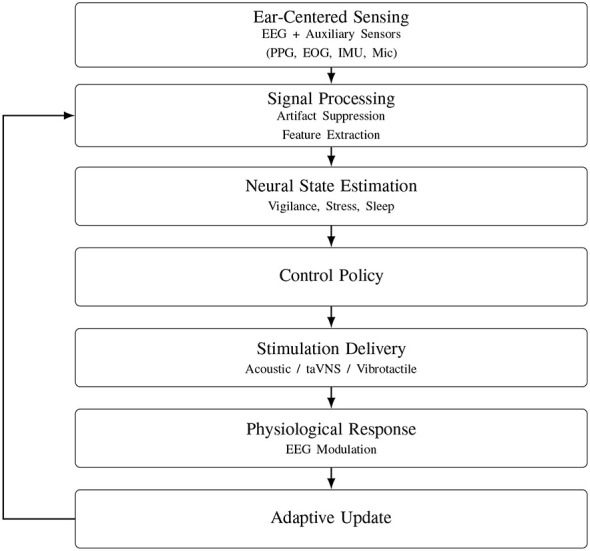
Conceptual closed-loop architecture for stimulation-integrated ear-EEG systems. Ear-centered EEG is combined with auxiliary sensing to estimate neural state and adapt stimulation parameters in real time.

Figure 13 illustrates a closed-loop architecture specific to stimulation-integrated ear-EEG systems. In contrast to open-loop earables, neural activity recorded at the ear directly informs stimulation control, enabling adaptive neuromodulation under real-world conditions. Despite conceptual appeal, most existing systems lack co-designed control policies, artifact-aware feedback pathways, and long-term stability guarantees, highlighting a major gap between proof-of-concept demonstrations and clinically deployable platforms.

From a circuits-and-systems perspective, this integration requires ultra-low-power acquisition, on-device processing, and precise stimulation drivers coexisting within sub-2 cm^3^ form factors. Figure 13 illustrates a generic closed-loop workflow: physiological and environmental signals are acquired, processed locally or with edge intelligence, translated into stimulation patterns, and continuously refined through user feedback.

Despite encouraging progress, critical challenges persist: improving sensor accuracy under noisy conditions, synchronizing multimodal data streams, and optimizing control policies for long-term stability. Addressing these barriers will be essential to fully realize the potential of earables as bioelectronic therapeutic platforms. Ultimately, such integration can enable naturalistic personalized interventions for stress, neurorehabilitation, and drug-resistant neurological pathologies.

## 5 Brain state monitoring and clinical applications of ear-EEG

An analysis of the existing ear-EEG literature reveals a clear concentration of application domains. The majority of published studies focus on (i) sleep and drowsiness monitoring, (ii) long-term epilepsy and seizure detection, and (iii) cognitive and affective state assessment under naturalistic conditions. A smaller but steadily growing body of work explores ear-EEG in rehabilitation and neurofeedback contexts.

This distribution reflects the intrinsic strengths of ear-centered EEG systems, including unobtrusive long-term wear, mechanical stability during daily activities, and suitability for ambulatory and home-based monitoring. Accordingly, the following subsections review clinical and functional applications of ear-EEG in descending order of prevalence within the literature, while highlighting emerging directions where evidence remains limited but promising.

### 5.1 Drowsiness detection using in-ear devices

Sleep and vigilance monitoring represent the most mature application domain of ear-EEG. The mechanical stability of in-ear electrodes and user tolerance for overnight wear make ear-EEG particularly suitable for long-duration sleep staging and drowsiness detection.

Table 7 summarizes representative ear-EEG studies for sleep and drowsiness monitoring, highlighting device form factors, target tasks, and key performance outcomes.

**Table 7:** Representative ear-EEG studies for sleep and drowsiness monitoring.

References	Ear-EEG device type	Target task	Key findings
Nakamura et al. (2018)	Custom viscoelastic in-ear EEG with flexible electrodes	Drowsiness detection	Demonstrated automatic drowsiness classification using ear-EEG with accuracy exceeding 80%.
Ha and Yoo (2016)	EEG–NIRS ear-module SoC (ear-hook form factor)	Vigilance monitoring	Showed feasibility of multimodal ear-centered EEG/NIRS for detecting missed stimuli and reduced alertness.
Hong et al. (2018)	Ear-canal EEG combined with PPG and ECG	Drowsiness recognition	Improved drowsiness classification by fusing ear-EEG with cardiovascular biomarkers.
Schwendeman et al. (2022)	Wireless ear-EEG with dry electrodes	Drowsiness detection	Achieved over 90% classification accuracy under ambulatory conditions using user-generic models.
Kaveh et al. (2024)	Wireless dry-electrode ear-EEG	Real-world drowsiness monitoring	Demonstrated high accuracy (93.3%) and robustness across users in naturalistic settings.

Beyond classification accuracy, these studies collectively highlight several system-level properties that make ear-EEG particularly well suited for sleep and vigilance monitoring. First, the mechanical anchoring of in-ear electrodes enables stable signal acquisition during overnight wear and prolonged seated or mobile conditions, reducing electrode displacement relative to scalp EEG. Second, the tolerance of users to ear-worn devices supports multi-hour and multi-day monitoring, which is critical for capturing state transitions such as microsleeps, circadian effects, and gradual vigilance degradation.

Despite this maturity, open challenges remain. Existing studies primarily focus on epoch-level classification performance, while fewer works examine long-term calibration drift, inter-night variability, or robustness under naturalistic motion and acoustic interference. Addressing these factors will be essential for translating ear-EEG–based sleep and drowsiness monitoring from controlled studies to large-scale clinical and occupational deployment.

### 5.2 Seizure detection and epilepsy monitoring

One of the most clinically compelling applications of ear-centered EEG is long-term epilepsy monitoring under naturalistic conditions. Conventional scalp EEG is impractical for continuous outpatient use due to setup complexity and poor tolerability, whereas ear-EEG enables multi-day to multi-week recordings with minimal disruption to daily life, making it well suited for ambulatory seizure surveillance. In practice, seizure detection using ear-EEG involves continuous recording of neural activity followed by automated analysis of characteristic patterns such as rhythmic discharges, spike–wave complexes, spectral alterations, or abnormal synchronization associated with seizure onset.

The sensitivity of ear-EEG to epileptiform activity has been systematically investigated using biophysical modeling. Meiser et al. (2020) demonstrated that ear-centered electrodes exhibit highest sensitivity to temporal and inferior cortical sources, which are commonly implicated in focal-onset epilepsy. While reduced sensitivity to deep or distant generators was observed compared to scalp EEG, the study confirmed that clinically relevant epileptiform discharges remain detectable within the ear-EEG lead field.

Building on these findings, clinical studies have demonstrated the feasibility of seizure and interictal discharge detection using ear-EEG in real-world settings. Joyner et al. (2024) reported successful detection of focal-onset seizures using a standalone ear-EEG device during long-term outpatient monitoring, highlighting the potential of ear-EEG as an unobtrusive alternative for seizure detection outside the hospital environment.

Beyond seizure detection, ear-EEG has also been validated for epilepsy-related sleep assessment. Jørgensen et al. (2020) showed that ear-EEG–based sleep staging in epilepsy patients achieves substantial agreement with scalp EEG, supporting its use for nocturnal monitoring where epileptiform activity and sleep architecture are closely intertwined. Such capabilities are particularly relevant given the strong coupling between sleep state and seizure occurrence.

Recent work has further expanded the clinical relevance of ear-EEG to the detection of subclinical epileptiform activity. Musaeus et al. (2023) demonstrated that long-term outpatient ear-EEG monitoring can reveal epileptiform discharges in patients with neurodegenerative disorders, underscoring the value of prolonged, low-burden EEG acquisition for identifying pathological activity that may be missed during routine clinical assessments.

Collectively, these studies indicate that while ear-EEG does not replace high-density scalp EEG for precise source localization, it offers a favorable trade-off between signal fidelity, anatomical relevance, and long-term wearability. This positions ear-EEG as a promising platform for ambulatory seizure detection, epilepsy-related sleep monitoring, and extended surveillance of epileptiform activity in both epilepsy and broader neurological populations.

### 5.3 Cognitive state and emotion assessment

Several studies have explored the use of ear-EEG for emotion recognition and cognitive-state assessment. Athavipach et al. (2019) demonstrated that ear-EEG signals recorded from temporal regions can be used to classify emotional valence and arousal, establishing the feasibility of affective decoding using compact, in-ear electrodes. More recent deep-learning approaches have further improved decoding performance by exploiting ear-EEG–specific feature representations under controlled experimental conditions (Mai et al., 2023, 2024).

Although classification accuracy remains lower than that achieved with high-density scalp EEG, ear-EEG offers key practical advantages—most notably unobtrusive wearability and suitability for prolonged, real-world monitoring. However, the current literature is largely limited to short laboratory sessions and offline analysis, with minimal investigation into cross-session stability, inter-subject generalization, or robustness under motion and daily-life conditions.

From a signal-processing perspective, the reduced spatial diversity of ear-EEG shifts the emphasis away from spatial feature selection toward temporal dynamics, cross-frequency coupling, and adaptive normalization strategies. Future research should therefore move beyond static emotion classification toward continuous cognitive-state tracking, focusing on models that explicitly account for context, fatigue, and individual variability. Addressing these challenges is essential for translating ear-EEG–based affective computing from proof-of-concept demonstrations into practical mental-health and human–computer interaction applications.

### 5.4 Rehabilitation and therapeutic applications of ear-EEG

Compared to sleep and epilepsy monitoring, rehabilitation-oriented applications of ear-EEG remain relatively underexplored. Existing studies primarily employ ear-EEG as a monitoring modality or as an auxiliary feedback signal within adaptive stimulation or motor–cognitive paradigms, rather than as a primary therapeutic control interface.

Recent work combining ear-EEG with vagus nerve stimulation or other closed-loop architectures suggests potential relevance for neurorehabilitation and plasticity-driven recovery. However, relatively few studies explicitly position ear-EEG as a rehabilitation-enabling technology, and most reported systems remain exploratory in nature. This highlights a clear gap between demonstrated sensing capabilities and their systematic integration into clinically validated rehabilitation workflows.

### 5.5 Neurofeedback and closed-loop control using ear-EEG

Neurofeedback represents a compelling application domain for ear-centered EEG, as it relies on continuous monitoring of brain rhythms and their transformation into real-time feedback signals that enable voluntary modulation of neural activity. While neurofeedback has traditionally been implemented using multichannel scalp EEG systems in laboratory or clinical environments, recent advances in ear-EEG raise the possibility of extending such paradigms to long-duration, mobile, and home-based settings.

Several studies have established that ear-EEG can reliably capture neural features commonly used in neurofeedback paradigms. In particular, modulation of alpha and mu rhythms, as well as event-related desynchronization and synchronization (ERD/ERS) associated with motor imagery, has been demonstrated using ear-centered electrode configurations. Although spatial coverage is more limited than scalp EEG, sensitivity to temporal and sensorimotor rhythms has been shown to be sufficient for detecting relative changes in band power and task-induced neural dynamics. These properties support the use of ear-EEG as a control signal source for feedback tasks based on rhythm up- and down-regulation rather than precise source localization.

The compact form factor and mechanical stability of in-ear electrodes further enable continuous recording over extended periods, reducing setup complexity and improving user compliance compared with conventional EEG caps. This makes ear-EEG particularly attractive for neurofeedback applications that depend on repeated or prolonged training sessions, such as stress regulation, attention training, or motor imagery rehearsal.

Beyond replicating existing neurofeedback paradigms, ear-EEG introduces capabilities that are difficult to achieve with scalp-based systems. Continuous, unobtrusive wear enables long-duration neurofeedback sessions spanning hours or days, opening opportunities for slow-timescale learning and behavioral conditioning. In mobile contexts, ear-EEG can support neurofeedback during walking, daily activities, or sleep, enabling feedback that adapts to vigilance state, fatigue, or circadian phase.

In rehabilitation settings, ear-EEG may facilitate home-based neurofeedback for motor recovery by coupling motor imagery–related ERD/ERS with auditory or vibrotactile cues, potentially reinforcing sensorimotor reorganization outside the clinic. Similarly, alpha-band modulation detected via ear-EEG could be used to drive adaptive auditory feedback for stress reduction or cognitive load management during real-world tasks. These use cases leverage the ear's suitability for simultaneous sensing and feedback delivery while maintaining minimal intrusion into daily life.

Despite these promising directions, neurofeedback using ear-EEG remains at an early stage, with several fundamental challenges yet to be addressed. First, there is no consensus on which neural features are most robust for ear-EEG–based feedback, with studies variably employing band power, ERD/ERS metrics, entropy measures, or hybrid features (Table 8). Feature stability across sessions and users remains poorly characterized, particularly under long-term wear and real-world conditions.

**Table 8:** Candidate neurofeedback control signals and their feasibility with ear-EEG systems.

Control signal	Typical neurofeedback use	Feasibility with ear-EEG (practical considerations)	Representative evidence
**Alpha-band power** (8–12 Hz)	Relaxation training, arousal regulation, and vigilance feedback	Highly compatible with low channel-count systems; robust bandpower estimation feasible. Sensitive to jaw and motion artifacts; benefits from artifact-aware preprocessing and baseline normalization.	Alpha modulation and reliable detection demonstrated in ear and ear-adjacent EEG studies (Mandekar et al., 2022; Looney et al., 2012; Moumane et al., 2024).
**SSVEP amplitude** (stimulus frequency)	Discrete BCI control, attention and engagement feedback	High SNR even with limited electrodes when visual flicker is present. Requires controlled visual stimuli; less suitable for eyes-free or naturalistic tasks.	SSVEP-based BCI feasibility demonstrated with ear-EEG and ear-adjacent configurations (Kondo and Tanaka, 2023).
**Mu/Beta ERD/ERS** (8–30 Hz)	Motor imagery, rehabilitation-oriented feedback	Theoretically relevant for rehabilitation, but spatial sensitivity of ear-EEG can limit robustness. Requires subject-specific calibration and conservative task design; few in-ear closed-loop demonstrations exist.	Spatial sensitivity limitations of ear-centered EEG for sensorimotor rhythms discussed in Meiser et al. (2020).
**ERP-based features** (e.g., P300)	Cognitive workload, attention feedback, oddball-based paradigms	Feasible in time-locked paradigms, but constrained by low SNR and limited channels. Mainly validated in scalp EEG; ear-EEG evidence remains limited.	ERP neurofeedback and workload markers reviewed in scalp EEG literature (Ghani et al., 2020).
**Sleep-stage/vigilance proxies** (spectral ratios, slow trends)	Sleep-adaptive cues, fatigue and vigilance monitoring	Well matched to ear-EEG strengths (long-duration wear, stability). Requires drift-aware thresholds and robust long-term artifact handling.	Sleep-related spectral features and signal stability validated using ear-EEG (Moumane et al., 2024; Zibrandtsen et al., 2016).

Evidence is strongest for spectral state markers (e.g., alpha-band power) and stimulus-locked responses; other candidates remain conceptually promising but underexplored in ear-EEG–specific closed-loop implementations.

Second, calibration drift poses a significant challenge for closed-loop ear-EEG neurofeedback. Changes in electrode–skin impedance, ear-canal geometry, and motion-related artifacts can alter signal characteristics over time, potentially degrading feedback accuracy if not explicitly modeled or compensated. Third, most existing demonstrations remain confined to controlled environments, with very few studies validating closed-loop ear-EEG neurofeedback during naturalistic tasks such as gait, daily activity, or unsupervised home use.

Finally, standardized protocols for ear-EEG neurofeedback—including task design, control features, feedback modalities, and performance metrics—are currently lacking. Addressing these gaps is essential for translating ear-EEG neurofeedback from proof-of-concept demonstrations into reliable, clinically meaningful interventions.

Overall, ear-EEG–based neurofeedback represents a largely untapped research direction that bridges brain-state monitoring, closed-loop control, and wearable neurotechnology. Its successful development could enable a new class of adaptive, real-world neurofeedback systems that extend beyond the limitations of traditional scalp-based approaches.

## 6 Embedded and edge intelligence in ear-EEG systems

**Figure 14:**
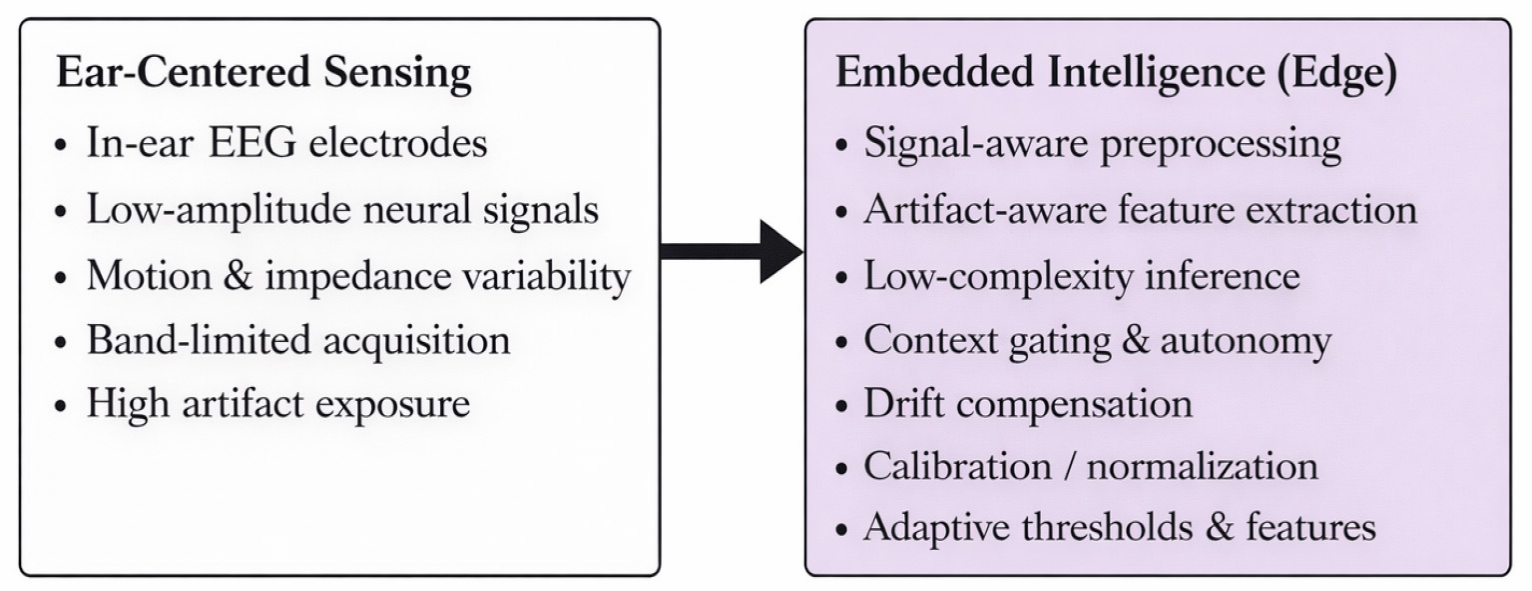
Embedded intelligence architecture for ear-EEG systems. Unlike generic TinyML pipelines, ear-EEG edge intelligence prioritizes signal-aware autonomy under long-term physiological drift and extreme power/memory constraints.

Embedded intelligence (EI) plays a pivotal role in enabling ear-centered EEG systems to transition from passive biosignal recorders into autonomous, adaptive, and clinically actionable platforms. In the context of ear-EEG, EI refers specifically to on-device or edge-level processing pipelines that operate under severe constraints on power, memory, latency, and form factor, while maintaining sufficient fidelity for neural signal analysis. Embedded intelligence refers to general on-device signal processing and inference capabilities, whereas embedded medical intelligence denotes clinically validated algorithms intended for diagnostic or therapeutic purposes. Current ear-EEG systems primarily implement the former, with clinical deployment requiring additional validation, reliability guarantees, and regulatory approval. Unlike generic wearable AI systems, ear-EEG imposes unique challenges due to the low signal-to-noise ratio of in-ear EEG, the need for continuous operation over extended periods, and the tight integration of sensing, processing, and wireless communication within a compact ear-worn form factor. Embedded intelligence in ear-EEG is not primarily about deploying deep learning models, but about enabling signal-aware autonomy under long-term physiological drift, motion variability, and extreme resource constraints, as conceptually illustrated in Figure 14.

### 6.1 Motivation for embedded intelligence in ear-EEG

The motivation for embedded intelligence in ear-EEG systems is fundamentally different from that of consumer audio wearables. Ear-EEG applications—including long-term seizure monitoring, vigilance assessment, cognitive-state tracking, and closed-loop neurofeedback—require continuous neural sensing with minimal latency and high reliability. Reliance on cloud-based processing is often impractical due to privacy concerns, intermittent connectivity, energy overhead associated with wireless transmission, and the need for real-time feedback in clinical and rehabilitation contexts.

Several ear-EEG studies have therefore emphasized local processing as a key system requirement. For example, Ha and Yoo (2016) introduced an EEG–NIRS ear-module system-on-chip (SoC) that performs signal acquisition, preprocessing, and vigilance-related feature extraction entirely on-chip, demonstrating that embedded processing is feasible even within strict power budgets. Similarly, Schwendeman et al. (2022) and Kaveh et al. (2024) highlight that transmitting raw ear-EEG continuously is energetically inefficient, motivating on-device feature extraction and classification. As a result, embedded intelligence in ear-EEG systems must be evaluated not only by inference accuracy, but by long-term stability, energy efficiency, and robustness to physiological and contextual variability.

### 6.2 Embedded processing pipelines in existing ear-EEG systems

Unlike generic TinyML benchmarks, embedded intelligence in ear-EEG systems is typically implemented as lightweight, task-specific pipelines rather than deep end-to-end neural networks. Common processing stages include band-limited filtering, artifact-aware feature extraction, and low-complexity classifiers operating on sliding windows of ear-EEG data.

Early examples include Nakamura et al. (2018), who implemented automated drowsiness detection using ear-EEG features suitable for low-power embedded deployment. Similarly Ha and Yoo (2016) further demonstrated the integration of EEG and NIRS processing on a single ear-mounted SoC, explicitly addressing latency and power constraints inherent to ear-worn devices.

More recent work has begun to incorporate embedded machine learning directly into ear-level platforms. Mai et al. (2023, 2024) demonstrated real-time emotion recognition using a behind-the-ear EEG device with on-device inference, emphasizing compact feature representations and efficient classifiers tailored to ear-EEG signals. An et al. (2025) presented a single-ear EEG device enabling real-time gesture recognition and interaction, illustrating how embedded inference can support closed-loop, user-interactive applications at the ear.

Importantly, these systems do not rely on large convolutional architectures common in vision tasks. Instead, they employ shallow networks, linear classifiers, or carefully constrained deep models designed around the statistical properties of ear-EEG rather than generic datasets such as ImageNet.

### 6.3 Hardware–algorithm co-design for ear-EEG embedded intelligence

A defining characteristic of embedded intelligence in ear-EEG is the need for tight hardware–algorithm co-design. Ear-EEG signals exhibit lower amplitude and reduced spatial diversity compared to scalp EEG, placing greater emphasis on temporal features, normalization strategies, and robustness to motion-induced artifacts. Consequently, EI in ear-EEG systems prioritizes deterministic latency, predictable memory usage, and continuous operation over peak inference accuracy.

Several platforms illustrate this principle. The wireless multi-electrode ear-EEG system by Kaveh et al. (2019) and Kaveh et al. (2024) integrates low-noise analog front-ends with embedded digital processing optimized for long-term use. Xu et al. (2023) introduced an in-ear multimodal sensor array capable of continuous brain-state and biochemical monitoring, explicitly designed to operate under edge-processing constraints where local computation minimizes wireless data transfer. Emerging work such as Sheeraz et al. (2024) further demonstrates closed-loop ear-worn EEG systems with real-time impedance monitoring and adaptive processing, reinforcing the role of embedded intelligence as a stability and reliability mechanism rather than merely a classifier. Table 9 summarizes ear-EEG systems that explicitly report embedded or edge intelligence, highlighting that most implementations rely on task-specific signal processing pipelines rather than fully adaptive on-device learning.

**Table 9:** Ear-EEG systems reporting embedded or edge intelligence, summarizing on-device processing strategies, system-level contributions, and reported limitations.

References	Ear(-EEG) platform	Embedded/edge intelligence strategy	Key contribution and limitations
Ha and Yoo (2016)	EEG–NIRS ear-module SoC	Fully on-chip DSP for preprocessing and vigilance-related feature extraction	Demonstrated feasibility of ultra-low-power, fully embedded processing; no adaptive learning or long-term drift compensation.
Nakamura et al. (2018)	Custom viscoelastic in-ear EEG	Low-complexity feature extraction with SVM-based drowsiness classification	Designed for embedded deployment; models trained offline with no on-device adaptation.
Schwendeman et al. (2022)	Wireless dry-electrode ear-EEG	On-device feature extraction with lightweight classifiers	User-generic models evaluated; continuous learning and calibration drift not addressed.
Kaveh et al. (2024)	Wireless dry-electrode ear-EEG	Edge-optimized preprocessing and classification to reduce wireless bandwidth	Robust performance in naturalistic settings; learning and adaptation remain off-device.
Mai et al. (2023)	Behind-the-ear EEG wearable	Real-time embedded ML inference using compact models	On-device emotion recognition demonstrated; evaluated in controlled laboratory sessions.
Mai et al. (2024)	Ear-EEG	Efficient deep-learning inference using ear-EEG-specific representations	Improved decoding accuracy with constrained models; no energy–accuracy trade-off analysis.
An et al. (2025)	Single-ear EEG device	Embedded inference for real-time interaction and gesture recognition	Demonstrated closed-loop interaction; long-term autonomy and stability not evaluated.
Xu et al. (2023)	Multimodal in-ear EEG–sweat platform	Edge processing to minimize wireless data transmission	Focus on sensing integration; neural decoding and intelligence largely exploratory.
Sheeraz et al. (2024)	Ear-worn EEG with impedance sensing	Closed-loop on-device impedance-aware processing	Improved signal reliability through real-time ESI monitoring; no task-level neural decoding.
Correia et al. (2024)	Ear-level EEG sensors	Embedded signal quality assessment and validation toolkit	On-device validation metrics for ear-EEG reliability; not a full decoding pipeline.

### 6.4 Limitations of current embedded intelligence approaches

Despite rapid progress in ear-EEG hardware miniaturization and signal quality, there remain remarkably few peer-reviewed studies that demonstrate fully embedded intelligence or on-device learning specifically for ear-EEG systems. Most existing implementations either stream raw or lightly processed signals off-device, or rely on edge-optimized preprocessing with classification and adaptation performed externally. As a result, truly autonomous ear-EEG systems capable of long-term operation, self-calibration, and adaptive inference remain largely unrealized. Despite significant advances in low-power electronics and wireless optimization, power consumption remains a key constraint due to the limited battery capacity of ear-worn devices.

Moreover, few studies systematically report the trade-offs between computational complexity, power consumption, and neural decoding performance, making cross-platform comparison difficult. This contrasts with the extensive benchmarking common in generic TinyML literature, highlighting the need for ear-EEG–specific evaluation frameworks.

### 6.5 Future directions: toward intelligent ear-EEG systems

Future embedded intelligence for ear-EEG systems will likely move beyond static inference toward adaptive, context-aware processing. Promising directions include artifact-aware feature selection driven by motion sensors, incremental calibration updates during long-term wear, and hierarchical processing architectures in which simple on-device models gate more complex processing only when needed.

Critically, progress in this area will require abandoning generic deep learning benchmarks in favor of ear-EEG–specific intelligence metrics that reflect clinical relevance, long-term stability, and energy efficiency. Embedded intelligence should therefore be viewed not as an optional enhancement, but as a foundational requirement for scalable, real-world deployment of ear-EEG systems in clinical monitoring, rehabilitation, and cognitive health applications. These capabilities are particularly critical for closed-loop neurofeedback and rehabilitation scenarios, where embedded intelligence must continuously adapt control signals under real-world conditions.

## 7 Emerging trends and future directions

As in-ear EEG wearables continue to evolve, it is crucial to examine the emerging trends that are shaping the future of this field. These trends focus on integrating multimodal sensing with edge-based ML algorithms, driving more sophisticated, personalized, and adaptive devices for real-time cognitive and physiological monitoring. Several key developments are expected to redefine the capabilities of in-ear wearables, especially in the domains of smart healthcare, neurorehabilitation, and continuous cognitive assessment. A significant trend is the integration of multimodal sensing capabilities into in-ear wearables. Although EEG monitoring has been the core of such devices, future wearables will combine EEG with other physiological signals, such as ECG, EMG, EOG, SpO2, sweat biomarkers, motion sensing (IMU), and even sweat glucose levels. This cross-modal data fusion enables a more comprehensive understanding of an individual's brain activity, autonomic responses, and overall health. Recent studies have shown that multimodal wearables can outperform single-modality devices by providing richer data sets that capture dynamic interactions between brain and body functions. Recent work suggests that combining EEG with additional physiological sensing—such as sweat-based biomarkers—may provide complementary insights into metabolic and cognitive state changes during task performance (Xu et al., 2023; Gao et al., 2018). Such multimodal sensing could support future closed-loop neurofeedback systems by enriching context-aware feedback. In the future, it is expected that these multimodal systems will allow continuous, unobtrusive monitoring of both brain activity and physiological parameters, facilitating more accurate and real-time assessments.

The integration of tiny ML algorithms directly on in-ear wearables is another breakthrough technology. As wearables become increasingly compact, they must also become computationally efficient to run sophisticated ML models that can process multimodal data in real-time. Edge computing advancements have made it feasible to integrate low-power ML models capable of performing feature extraction, pattern recognition, and classification tasks on the device itself, without requiring cloud connectivity. Research has shown that MCUs can support the execution of lightweight CNNs or RNNs, even in small-form factor devices such as in-ear wearables. These low-latency, on-device models can analyze incoming EEG signals to detect cognitive states, such as attention or relaxation, while simultaneously processing motion signals or sweat biomarkers to provide personalized interventions.

One of the foremost challenges in in-ear wearables is power consumption, given the small battery capacity of these devices. However, the advent of energy-efficient ML algorithms and energy-harvesting technologies has paved the way for more sustainable operation. Energy harvesting from the natural motion of the ear canal, such as jaw movement or head rotation, presents a promising solution to power wearables without relying on external charging. Early prototypes have shown that up to 20μW of power can be harvested from ear canal motion, sufficient for low-power sensor operations. Furthermore, power-efficient ML models, specifically designed for low-energy MCUs, are being developed to minimize energy usage while maintaining the ability to execute complex real-time analysis. The use of quantized neural networks, low-rank approximations, and pruning techniques can further optimize these models to run on limited power without sacrificing performance. These advances are essential to enable 24/7 wearability, facilitating continuous brain and body monitoring without significant intervention. Another key trend is the development of context-sensitive adaptive wearables. Future in-ear devices will be capable of dynamically adjusting their functionality based on continuous analysis of both cognitive states and environmental conditions. Reinforcement learning (RL) algorithms could be employed to create self-optimizing systems that not only learn from user data, but also adapt their monitoring and stimulation approaches based on the real-time context. For example, in neurorehabilitation, a wearable system could use an RL algorithm to modify electrical or acoustic stimulation based on EEG signals or physiological parameters such as heart rate or muscle activity. Real-time feedback could be generated to adjust the intensity or frequency of stimulation depending on the user's progress and cognitive state, allowing personalized therapy to evolve as individual needs change. Future applications of these devices could support patients with stroke recovery, cognitive impairment, or attention disorders, providing customized interventions for more effective rehabilitation.

As in-ear EEG wearables become more interconnected and capable of collecting sensitive physiological and cognitive data, data privacy and security will be critical. In the near future, advanced encryption techniques will be required to safeguard personal data, particularly in the context of healthcare applications. Emerging trends suggest the use of blockchain-based systems or federated learning models, which allow for secure, decentralized data processing, and ML without compromising user privacy. In addition, secure edge computing frameworks, where data analysis occurs locally on the device, can further minimize the risks associated with transmitting sensitive information to the cloud. As wearables continue to evolve, the development of privacy-preserving algorithms will be vital to ensure user confidentiality and regulatory compliance.

## 8 Bias considerations in reviewed studies and device limitations

While this review focuses mainly on the technical development and applications of in-ear EEG systems, we acknowledge that potential biases may exist in the included literature. Alfalahi et al. (2022) and Alhussein et al. (2022) noted that wearable neurotechnology studies can be affected by selection bias, reporting bias, and limited standardization across experimental settings. Furthermore, geographical overrepresentation, particularly from Europe and East Asia, and underreporting of negative or inconclusive outcomes can influence the perceived efficacy and generalizability of in-ear EEG systems. Although a formal bias assessment was not feasible due to the large number of studies included, this review adopts a neutral interpretive position and highlights the importance of addressing these methodological concerns in future standardized evaluations.

Regarding technological limitations, in-ear EEG devices often exhibit lower signal quality compared to scalp EEG, reduced spatial coverage due to fewer electrodes, and are more susceptible to motion artifacts. In addition, device fit and user comfort can vary significantly between individuals. Although these limitations have been discussed throughout the manuscript in sections on electrode design, signal acquisition, and validation, they remain key challenges for future refinement and the wider adoption of in-ear neurotechnology.

## 9 Conclusions

This literature review provides a comprehensive and structured overview of EEG technologies in the ear, addressing several key limitations found in previous reviews. Unlike past work, we propose a unified taxonomy that connects device design, signal acquisition methods, processing pipelines, and application domains, offering a clearer framework for researchers and developers. We also present a detailed analysis of artifact sources and mitigation strategies, highlighting the need for standardized metrics in signal quality evaluation. Furthermore, this paper systematically compares spatial sensitivity and source coverage across in-ear configurations, a topic previously underexplored. In contrast to earlier studies focusing solely on linear metrics, we advocate for the integration of non-linear signal similarity measures to enhance future analyses. In doing so, this survey not only synthesizes the current state of in-ear EEG, but also sets the foundation for more informed, standardized, and innovative development in this evolving field. Based on more than 87 published studies, we have described the wide variety of applications that in-ear wearable offers, from detecting a user's physiological state under different conditions (drowsiness, stroke, or emotion) to various innovations in rehabilitation. We have also discussed how combining sensing and stimulation processes offers a complacent, non-invasive, and socially acceptable method of administering therapeutic therapies. In-ear wearable devices have great potential for neuromodulation, but integration of embedded intelligence—leveraging AI-driven adaptive algorithms, real-time monitoring, and personalized stimulation—will be crucial to optimize their efficacy, precision, and impact on neurological rehabilitation, including stroke recovery. We emphasize that future research should focus on developing smart earbuds capable of sensing multimodal signals while integrating stimulation techniques with embedded intelligence, enabling more precise, adaptive, and personalized therapeutic applications.
